# Encapsulation of Capsaicin in Oil‐In‐Water Nanoemulsion: Optimization by a Mixture Design and Its Application in Merguez Sausage Preservation

**DOI:** 10.1002/fsn3.70042

**Published:** 2025-02-23

**Authors:** Eya Soussi, Khouloud Rigane, Anis Ben Hsouna, Miroslava Kačániová, Wissem Mnif, Zaina Algarni, Moncef Chouaibi

**Affiliations:** ^1^ Higher School of Food Industries of Tunisia University of Carthage ElKhadra City, Tunis Tunisia; ^2^ Laboratory of Biotechnology and Plant Improvement Centre of Biotechnology of Sfax Sfax Tunisia; ^3^ Department of Environmental Sciences and Nutrition, Higher Institute of Applied Sciences and Technology of Mahdia University of Monastir Monastir Tunisia; ^4^ Institute of Horticulture, Faculty of Horticulture Slovak University of Agriculture Nitra Slovakia; ^5^ Department of Bioenergy, Food Technology and Microbiology, Institute of Food Technology and Nutrition University of Rzeszow Rzeszow Poland; ^6^ Department of Chemistry, College of Sciences at Bisha University of Bisha Bisha Saudi Arabia; ^7^ Department of Physics, Faculty of Sciences at Bisha University of Bisha Bisha Saudi Arabia

**Keywords:** capsaicin, merguez preservation, mixture design, O/W nanoemulsion, physicochemical properties, shelf life estimation

## Abstract

In this research work, a mixture design was applied to optimize the encapsulation of capsaicin in oil–water‐nanoemulsion using almond gum, pea protein isolate, and citrus pectin as independent variables. Therefore, results indicated that the cubic, special cubic, and quartic models were the most adequate to describe the variation of responses as a function of independent variables. Therefore, the pea protein isolate showed the highest effect on mean droplet size, polydispersity index, ξ‐potential, and antioxidant activities. However, the almond gum was the most significant component in whiteness and plastic viscosity (*p* < 0.05). Moreover, the rheological properties of the nanoemulsions demonstrated that are non‐Newtonian fluids with pseudoplastic (shear thinning) behavior and are well‐fitted by the Casson model. Remarkably, all the nanoemulsions exhibited antioxidant activities, in which the almond gum and pea protein isolate combination indicated the highest activities (IC_50_). Also, the formulated nanoemulsions exhibited more sensitivity to the Gram‐positive bacteria ( *Listeria monocytogenes* and *Staphylococcus aureus* ). Thus, the statistical data revealed that the mixture of almond gum (16.13%), pea protein isolate (73.45%), and citrus pectin (10.42%) was proven to be the optimum condition. Besides, these findings indicated that there are no significant differences between optimal conditions and those obtained in practice (*p* > 0.05). Under these conditions, the experimental values of mean droplet size, polydispersity index, ξ ‐potential, whiteness index, plastic viscosity, creaming index, encapsulation yield, and the inhibitory concentration at 50% were 3.58 nm, 12.13%, −30.53 mV, 78.65, 0.12 Pa·s, 1.96%, 94.06%, and 23.01 μg/mL, respectively. Furthermore, encapsulated capsaicin was used to Merguez preservation and the results revealed that pH, color parameters, TVB‐N, TBARS amounts, textural properties, and their shelf life were improved over a storage time of 30 days at 4°C. Hence, the findings are encouraging and allow considering the use of capsaicin nanoemulsions based on almond gum for the enrichment of food, cosmetic, and pharmaceutical products.

## Graphical abstract

Our findings are encouraging and allow considering the use of capsaicin nanoemulsions based on almond gum for the enrichment of food, cosmetic, and pharmaceutical products.

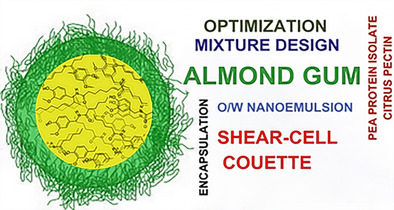

## 1 Introduction

Pepper is one of the most important vegetables cultivated in Africa, Asia, America, and the Mediterranean regions (Chouaibi, Mejri, et al. 2019; Chouaibi, Rezig, et al. 2019). Interestingly, capsaicin (trans‐8‐methyl‐N‐vanillyl‐6‐nonenamide) is the abandoned bioactive substance found in seeds, responsible for the spicy flavor (Chouaibi, Mejri, et al. 2019; Chouaibi, Rezig, et al. 2019; Wu et al. 2022). In addition, it is a natural alkaloid known as chili fruit (Hudita et al. 2021). It constitutes the main capsaicinoid compound derived from plants of the genus Capsicum as demonstrated by Romano et al. (2021). In recent years, capsaicin has occupied an important place in the food, chemical, cosmetic, and medical industries (Wang et al. 2021). Interestingly, several studies have well‐documented the beneficial effects of capsaicin on human health (Isaschar‐Ovdat et al. 2021). It is an antioxidant compound that has anti‐inflammatory, cardio‐protective, anti‐carcinogenic, and antimicrobial activities (Isaschar‐Ovdat et al. 2021; Wang et al. 2021). However, despite its beneficial effects, recent studies have shown that the ingestion of capsaicin can generate adverse effects at high concentrations on digestion and metabolism with gastrointestinal disease due to the high biting power (Xiang et al. 2021). In addition, capsaicin is a hydrophobic compound that is characterized by high instability, low bioavailability, and water solubility, as well as reduced durability at temperature and pH, which limit its use (Wu et al. 2022; Sánchez‐Arreguin et al. 2019; Akbas et al. 2018). Thus, some studies have proposed mechanisms based on encapsulation (Xiang et al. 2021).

Nanoencapsulation is a technique that can be performed by physical, chemical, or physicochemical methods (Chouaibi, Mejri, et al. 2019; Chouaibi, Rezig, et al. 2019; Shahid et al. 2021; Snoussi et al. 2022; Telmoudi et al. 2023). It allows the formation of nanocapsules at the micrometric or nanoscale containing solid, liquid, and gaseous active ingredients and protected by a layer of encapsulation materials (Shahid et al. 2021). However, nanoencapsulation has been developed in the cosmetic, medicine, pharmaceutical, and food industries (Chouaibi, Mejri, et al. 2019; Chouaibi, Rezig, et al. 2019). It makes it possible to delay the start of oxidation and hydrolysis of bioactive compounds during treatments, thus, maintaining their stability and improving their bioavailability with the masking of bad tastes and astringencies of polyphenols (Shahid et al. 2021). In the form of microcapsules, the pungent and irritating effect of capsaicin on the mouth, esophagus and stomach disappears (Günel et al. 2021). Nanoemulsions are one of the strategies that have been studied to improve the bioavailability of capsaicin (Lu et al. 2020). They are colloidal dispersions at the nanoscale and have high stability and bioaccessibility as well as encapsulation efficiency of capsaicin, and improve biological activities such as antioxidant and anti‐inflammatory activities (Akbas et al. 2018; Lu et al. 2020).

Tree gums are renewable biopolymers applied in nanoparticle formulations such as almond gum which is produced by the trunk of the almond tree (*Purnis dulcis*) (Sree et al. 2022; Telmoudi et al. 2023). Exudate gums of trees are obtained due to certain stress produced by the gummosis process (Dhaka et al. 2021). Interestingly, almond gum is composed mainly of carbohydrates consisting of arabinose, galactose, xylitol, and uronic acid with traces of glucose, rhamnose, and mannose as well as minerals such as potassium, sodium, magnesium, iron, and calcium as reported by Mahfoudhi et al. (2012). The amount of these components is varied according to the season, and the region of cultivation, as well as the purification method, and used solvent (Mahfoudhi et al. 2012). This gum has important biological, chemical, and physical properties (Dhaka et al. 2021). Almond gum nanofibers can be used as a carrier to increase the solubility of hydrophobic compounds, which facilitates their use in pharmaceutical, cosmetic, and food industries (Rezaei and Nasirpour 2018).

Several researchers have studied the influence of combining different wall materials on stability and this combination has given better protection compared to a single wall material (Labuschagne 2018). The protein‐polysaccharide combination provides novel biopolymer complexes such as protein isolate and polysaccharide interactions which are used for the perception of different types of colloidal systems to enhance the physical and chemical stability of double emulsions, nanoparticles, and microparticles (Wusigale Liang and Luo 2020; Ma and Chatterton 2021; Snoussi et al. 2020).

In the present work, we will prepare capsaicin‐oil‐in‐water nanoemulsions based on experimental design and stabilized by pea protein isolate, almond gum, and citrus pectin allowing durable encapsulation of capsaicin. We will also detail the technical characterization of these nanoemulsions, such as mean particle size, ξ‐potential, transparency color, optical microscopy observation, creaming and polydispersity indexes, the antioxidant and antibacterial activities, as well as the rheological properties of O/W nanoemulsions. The objective of this study is to assess the most suitable biopolymer and nanoemulsion formulation to produce effective stability of encapsulated capsaicin. Also, the formulated nanoemulsions were used to preserve the beef Merguez to extend their shelf life during cold storage.

## 2 Material and Methods

### 2.1 Chemical Reagents

1,1‐dephenyl‐2‐picrylhydrazyl (DPPH), methanol, ethyl acetate, dimethyl sulfoxide (DMSO), and pure capsaicin (≥ 95%) were purchased from Sigma Chemical Co. (Sigma‐Aldrich GmbH, Steinheim, Germany). Pea protein isolate (PPI, 99%) was obtained from Xi'an Zelong Biotech Co. Ltd. (Xian, China). Citrus pectin (71.10% degree of methyl esterification and galacturonic acid > 65%) was acquired from MP Biomedical (Netherlands). Nutrient broth and agar were purchased from Biokar Diagnostics (Beauvais, France.) The sunflower oil was obtained from a local supermarket (Tunis, Tunisia). *Listeria monocytogenes* , *Staphylococcus aureus* , *Escherichia coli* , *Salmonella arizonae*, and *Pseudomonas aeruginosa* were obtained from the Higher School of Food Industries of Tunisia (ESIAT). Ultrapure distilled deionized water was generated from the water purification system (Medica 15 BP, ELGA LabWater, Woodridge, IL). All the other chemicals and solvents used in this study were purchased from Sigma Chemical Co. (Sigma‐Aldrich GmbH, Steinheim, Germany) and were of analytical grade.

### 2.2 Materials

Almond gum used in this work was collected manually by the authors from almond trees ( *Prunus dulcis* ) in March 2023 in Sidi Bouzid (Tunisia), and stored in a dry chamber (≈30°C). It was then ground using a mortar, and pestle. They were transferred into an electric blender (Moulinex, France) for further grinding to obtain a fine powder. Then, the obtained almond powder was mixed with ethanol ((90%), and 2/3 (w/v)). The mixture was centrifuged at 3000 *g* for 15 min using LMC‐centrifuge (LMC‐4200R, Biosan, Latvia). After, the obtained supernatant was lyophilized using a laboratory freeze‐dryer (Christ gamma 1‐16, LSC freeze‐dyer, Germany). Finally, the purified almond gum was stored at room temperature (≈22°C) until assays were performed. Sausages (beef Merguez) were purchased from supermarkets (Géant, Tunis, Tunisia). They were treated with the nanoemulsion at their first preparation day and were stored at a refrigerated temperature (4°C).

### 2.3 O/W Nanoemulsion Formulation

The oil‐in‐water (O/W) nanoemulsion was carried out according to the simplex lattice mixture design (Table 1). For the aqueous phase, almond gum, and pea protein isolate solutions were prepared at concentrations of 5 (w/w) in deionized water, and 1.5% in phosphate buffer (pH 7), respectively. Then, they mixed separately using a magnetic stirrer (IKA, Germany) for 10 h at 30°C. Likewise, the citrus pectin solution was prepared at a concentration of 1.50% (w/w) in ultrapure water, and mixed using the magnetic stirrer (150 rpm, 4 h, 60°C), then stored at a refrigerated temperature (4°C) for 24 h to ensure complete hydration. For the oil phase, 200 mg/kg of capsaicin was mixed in sunflower oil and homogenized until the mixture dissolved. Then, the oil phase was filtered using a Whatman paper filter (Sigma‐Aldrich, USA). The capsaicin nanoemulsions were prepared by adding 20% (w/w) of the oily phase into aqueous phase. Firstly, the two phases were then homogenized using Ultra‐Turrax mixer T25 (IKA Werke GmbH & Co., DE) at 2000 g for 2 min and were left in ambient air. This obtained O/W emulsion is then sheared in a shear‐cell “Couette” constituted of a coaxial rotor and stator, separated by a small air gap (concentric cylinder geometry, Ademtech SA, France). The small air gap makes it possible to apply spatially homogeneous shear and very high stresses. The applied shear speed is $\dot{\gamma }$ = 12,000 s^−1^ for 50 s. The obtained O/W nanoemulsions were stored, in glass tubes at obscurity, at 4°C for further analysis.

**TABLE 1:** The experimental design of the aqueous phase used to prepare the encapsulation of capsaicin in O/W nanoemulsion.

Formulations	Uncoded independent variables			Coded independent variables		
	Almond gum	Pea protein isolate	Citrus pectin	A	B	C
F1	5	0	0	1	0	0
F2	5	0	0	1	0	0
F3	0	1.50	0	0	1	0
F4	0	0	1	0	0	1
F5	2.50	0.75	0	0.50	0.50	0
F6	0	1.50	0	0	1	0
F7	0.83	0.25	0.67	0.167	0.167	66.67
F8	2.50	0	0.50	0.50	0	0.50
F9	0	0.75	0.50	0	0.50	0.50
F10	1.67	0.50	0.33	33.33	33.33	33.33
F11	3.33	0.25	0.167	66.67	16.67	16.67
F12	0	0	1	0	0	1
F13	0.83	1	0.167	16.67	66.67	16.67

*Note:* A: almond gum; B: pea protein isolate; C: citrus pectin, total concentrations used in nanoemulsion of almond gum, pea protein isolate, and citrus pectin were 5%, 1.50%, and 1% (w/w), respectively.

### 2.4 Simplex Lattice Design

Food nanoemulsions are thermodynamically unstable systems hence, the mixture design was applied to optimize the composition of the aqueous phase as well, as to evaluate the effect of the proportions of the mixing components on the studied parameters. In our design, three components were examined such as almond gum (A), pea protein isolate (B), and citrus pectin (C). This experimental design is presented in Table 1, and with all the proportions of the components (0%–100%), this tool optimizes the mixture and analyzes the interactions between its factors or independent variables. All compositions of the prepared nanoemulsions are shown in Table 1. The dependent variables or responses were: mean droplet size (d_[4,3]_) (nm), polydispersity index (PDI), ξ‐potential (mV), whiteness index, creaming index (%), plastic viscosity (Pa·s), yield encapsulation (%), antioxidant activities (IC_50_, μg/mL), and antibacterial activities (diameter inhibition, mm) of each bacteria ( *Listeria monocytogenes* , *Staphylococcus aureus* , *Escherichia coli* , *Salmonella arizonae*, and *Pseudomonas aeruginosa* ).

### 2.5 Mathematical Models

At each experimental point, the experimental data was fitted according to linear, quadratic, cubic, and special cubic models, as, respectively, described by Telmoudi et al. (2023):

(1) $$Y=\beta_{A}\times A+\beta_{B}\times B+\beta_{C}\times C$$

(2) $$\begin{array}{cc} Y= & \beta_{A}\times A+\beta_{B}\times B+\beta_{C}\times C+\beta_{\mathrm{AB}}\times A\times B+\beta_{\mathrm{AC}}\times A\times C+\\ & \beta_{\mathrm{BC}}\times B\times C \end{array}$$

(3) $$\begin{array}{cc} Y= & \beta_{A}\times A+\beta_{B}\times B+\beta_{C}\times C+\beta_{\mathrm{AB}}\times A\times B+\beta_{\mathrm{AC}}\times A\times C+\\ & \beta_{\mathrm{BC}}\times B\times C+\beta_{\mathrm{ABC}}\times A\times B\times C \end{array}$$

(4) $$\begin{array}{cc} Y= & \beta_{A}\times A+\beta_{B}\times B+\beta_{C}\times C+\beta_{\mathrm{AB}}\times A\times B+\beta_{\mathrm{AC}}\times A\times C+\\ & \beta_{\mathrm{BC}}\times B\times C+\delta_{\mathrm{AB}}\times A\times B\times \left(A-B\right)+\delta_{\mathrm{AC}}\times A\times C\times \left(A-C\right)+\\ & \delta_{\mathrm{BC}}\times B\times C\times \left(B-C\right)+\beta_{\mathrm{ABC}}\times A\times B\times C \end{array}$$

where *Y* is the estimated response; *β*_*A*_, *β*_*B*_, *β*_*C*_, *β*_*AB*_, *β*_*AC*_, *β*_*BC*_, *β*_*ABC*_, *δ*_*AB*_, *δ*_*AC*_ et *δ*_*BC*_ are the regression coefficients; *A*, *B*, and *C* are the proportions of the components almond gum, pea protein isolate, and citrus pectin, respectively.

### 2.6 Characterization of Nanoemulsions

#### 2.6.1 Oil Droplet Size, Polydispersity Index, and ξ‐Potential Determinations

A dynamic laser droplet size analyzer (Nano‐S, Malvern Instruments Ltd., Worcestershire, UK) was used to evaluate the diameter distributions (d_[4,3]_), the ξ‐potential, and the polydispersity index (PDI) of droplets. Helium–neon laser (4 mW) was fixed at 632.8 nm. One milliliter of each nanoemulsion was diluted in 50 mL of deionized water. The refractive index of water dispersant and oil were 1.33 and 1.46, respectively. Diameter distributions were calculated from the auto‐correlation function using the integrated analysis software and conducted in triplicate.

#### 2.6.2 Whiteness Index (WI)

Color measurements of O/W nanoemulsions were determined using a CR‐400 colorimeter (Konica Minolta Sensing Inc., Osaka, Japan). About 30 mL of each nanoemulsion was placed in glass Petri dishes of 60 mm diameter and 15 mm height under dark conditions to measure the tristimulus color coordinates (L*a*b*) represented by the CIE L*a*b* system. The L* indicates the lightness and varies, respectively, from black to white (0–100), the a* designs red (positive value) and green (negative value) colors, and the b* characterizes yellow (positive value) and blue (negative value) colors. The whiteness index (WI) was estimated using the following equation Equation (5) (Vargas et al. 2008):

(5) $$\mathrm{WI}=100-{\left({\left(100-L*\right)}^{2}+a^{*2}+b^{*2}\right)}^{0.5}$$

With L*, a*, and b* are the color coordinates for each formulation. Each sample was measured in triplicate.

#### 2.6.3 Creaming Index (CI) Determination

Creaming index (CI) was used to evaluate the stability of nanoemulsions, and 40 mL of each O/W nanoemulsion was placed directly after preparation into graduated test tubes (1 cm in diameter × 15 cm in height) and left for 15 days at 4°C. During this period, the nanoemulsions were separated into two layers. The first was at the top and constituted the cream layer, and the second was at the bottom and constituted the serum layer. These heights were measured after 15 days of cold storage and compared to that of the initial nanoemulsion to calculate the creaming index using Equation (9):

(6) $$\mathrm{CI}\left(\%\right)=\frac{H_{S}}{H_{T}}\times 100$$

where *H*_*S*_ and *H*_*T*_ are the heights of the serum layer and the total emulsion, respectively. The measurements were made in triplicates.

#### 2.6.4 Optic Microscopic Morphology Observation

The nanoemulsion was placed between a microscope slide and a cover slip. The microstructure of the nanoemulsion was observed with an optical microscope (WF10X, Olympus, Japan) connected to an HDCE‐50B digital camera (China) for image capture. These images were then processed by Micrometrics SE Premium v2.7.

#### 2.6.5 Rheological Behavior

The apparent viscosity of O/W nanoemulsions was determined using a Rheometer (MCR 102, Anton Paar, Austria) fitted with a coaxial concentric with a diameter of 25 mm. The shear rate varied from 0.01 to 500 s^−1^. The regulation of temperature was done using a circulator bath (Julabo GmbH, Germany), and fixed at 20°C (corresponding to the temperature inside the nanoemulsion). The rheometer was connected to computer software to analyze data. An aliquot of each O/W nanoemulsion was sheared in the gap between the fixed inner cylinder, and the rotated outer cylinder. Besides, rheological models were tested to describe the type of fluid and their behavior. For that, the shear stress and shear rate were adjusted according to the Power law (Ostwald) and Casson models as follows by Equations (7 and 8):

(7) $$\sigma =k\;{\dot{\gamma }}^{n}$$

(8) $$\sigma^{0.50}=\sigma_{c}^{0.50}+{\left(\mu_{c}\cdot \dot{\gamma }\right)}^{0.50}$$

where *σ* is the shear stress (Pa), *k* is the consistency coefficient (Pa·s^*n*^), *γ* is the shear rate (s^−1^), *n* is the flow behavior index (dimensionless), *σ*_*c*_ is the yield stress (Pa), and *μ*_*c*_ is the plastic viscosity (Pa·s).

#### 2.6.6 Yield of Capsaicin Encapsulation

The amount of capsaicin encapsulated in the O/W nanoemulsion was estimated according to the procedure of Gammoudi et al. (2021), with a slight modification. Briefly, 0.3 mL of each O/W nanoemulsion was mixed with 4.2 mL of ethyl acetate. Subsequently, the mixture was vortexed for 5 min and centrifuged (Hettich D‐78532, Tuttlingen, Germany) at 1400 g for 7 min. The supernatant was filtered through Whatman filter paper No. 1, then through a Millipore filter with a pore size of 0.22 μm. The capsaicin amount was quantified using a liquid chromatography‐mass spectroscopy system (LC‐MS), equipped with an autosampler, a degasser, and a quadruple mass spectrometer analyzer. The operating conditions were: mobile phase flow rate was 0.30 mL/min, the mobile phase consisted of water and formic acid with 0.30% (v/v) (phase A), and acetonitrile with a ratio of 0.3 (v/v) (phase B), an AQUASIL C18 column (150 mm × 3.0 mm) with a particle size of 3 μm was applied. A calibration plot was achieved using a commercial standard of capsaicin and 10 μL was injected into the HPLC apparatus for analysis. The detection was achieved by MS, and the analyses by an electrospray ionization (ESI), and an ion trap mass analyzer operating in positive mode and controlled by the software analysis. The obtained regression equation was *Y* = 23.40*x* + 80 with a determination coefficient of 0.98. Also, the limits of detection and quantification, and the recovery were 0.20 mg/kg, 0.10 mg/kg, and 0.96, respectively. The capsaicin yield (%) is dependent on the concentration of capsaicin, and the absorbance of nanoemulsion hence, it was estimated by the expression that follows, Equation (9):

(9) $$Y\left(\%\right)=\frac{C_{i}}{C}\times 100$$

where *Y* signifies the yield of capsaicin encapsulation (%), *C* denotes the total concentration of capsaïcin added in the O/W nanoemulsion (μg/mL) and *C*_*i*_ is the concentration of capsaicin in nanoemulsion at time t (μg/mL).

#### 2.6.7 Antioxidant Activities by DPPH Test

The free radical scavenging activities of O/W nanoemulsions were measured by the DPPH assay according to the method published by Telmoudi et al. (2023) with some modifications. Each encapsulated capsaicin was reacted with the stable DPPH radical in a methanol solution. Briefly, 0.5 mL of each nanoemulsion (where it was stored for 2 days under sunlight) was mixed with 3 mL of absolute methanol and 0.3 mL of DPPH radical solution (0.5 mM in methanol). When the color of the mixture changes from violet to yellow (reduction of DPPH by donating hydrogen by the encapsulated capsaicin), the absorbance was measured at 517 nm after 30 min of reaction by a UV–visible spectrophotometer (Jenway 6705 UV/Vis). About, 3.8 mL of methanol solution of DPPH was selected as a blank. The mixture of 3.5 mL of methanol and 0.3 mL of DPPH radical solution was mixed and utilized as a control solution. Hence, the anti‐radical activities of O/W nanoemulsions were calculated according to the following Equation (10):

(10) $$\text{Inhibition percentage}\;\left(\%\right)=\frac{A_{\text{control}}-A_{\text{sample}}}{A_{\text{control}}}\times 100$$

where *A*_control_ and *A*_sample_ were the absorbance values of the control as well as the tested sample, respectively. IC_50_ (μg/mL) represents the concentration of encapsulated capsaicin to reduce 50% of the free radicals of DPPH and was determined by plotting the inhibition graph percentage against concentrations. Therefore, SigmaPlot software (v.14, California, USA) was applied to calculate the inhibitory concentration (IC_50_) using non‐linear regression.

#### 2.6.8 Antibacterial Activities

The disc diffusion method (Kirby‐Bauer) was applied to evaluate the antibacterial activities of capsaicin nanoemulsions according to the method described by Lima et al. (2021). This test was applied on two Gram‐positive bacteria ( *Listeria monocytogenes* (ATCC 11120) and *Staphylococcus aureus* (ATCC 25923) and three Gram‐negative bacteria *Escherichia coli* (ATCC 25922), *Salmonella arizonae* (ATCC 25922), and *Pseudomonas aeruginosa* (ATCC 9027).

##### 2.6.8.1 Bacterial Cultures

To prepare the bacterial suspensions, 1 mL of each of the mentioned bacteria was taken and transferred into tubes containing nutrient broth and incubated at 37°C for 24 h. Therefore, the final bacterial concentration was assessed by measuring the optical density values at 600 nm using a spectrophotometer (Jenway 6305 spectrophotometer, UK).

##### 2.6.8.2 Agar Diffusion Method (Disc Diffusion Assay)

Briefly, 100 μL of the bacterial suspensions (1 × 10^7^ CFU/mL) were added in sterile Petri dishes that contained 20 mL of the Nutrient agar. Afterward, the sterile discs with a 6 mm diameter were immersed into the capsaicin nanoemulsions, placed on plates, and then incubated at 37°C for 18–24 h. In each Petri dish, four discs were applied. The first was a blanc disc without capsaicin nanoemulsion and DMSO (Dimethyl sulfoxide). The second was impregnated by the DMSO to confirm the non‐activities of selected bacteria. Hence, the third disc was impregnated only in capsaicin nanoemulsion and the last was used as positive control where it was impregnated in the gentamicin. After incubation, the data were obtained by measuring the inhibition zone diameter with a digital caliper (mm) and the sensitivity of the bacteria to the tested capsaicin nanoemulsions.

### 2.7 Application to Sausage (Merguez) Preservation

Merguez sausages are purchased from the local market (Géant, Ariana, Tunisia), and transported directly to the laboratory under aseptic conditions in polystyrene bags containing ice packs. All Merguez samples do not contain the pepper or its extracts. The Merguez samples are immersed directly (10 min) in the O/W nanoemulsions which are prepared according to the optimal conditions found by the mixture design. Thus, control does not undergo immersion in the O/W nanoemulsions, NM are immersed in nanoemulsions without capsaicin, CNM: the Merguez samples are treated with nanoemulsions containing encapsulated capsaicin. The Merguez samples are stored in a refrigerator at 4°C to monitor the physicochemical parameters during storage time. Each treatment is repeated 10 times using independent samples. For the microbiological analysis, the samples were stored at different temperatures of 4°C, 15°C, and 25°C.

#### 2.7.1 Determination of Physicochemical Parameters

The pH of Merguez samples was determined using Mettler Toledo (Mettler T oledoS210K, UK). The water loss was assessed by the mass variation of Merguez samples between the initial mass, and the mass at time t, using precise balance (Mettler Toledo, UK). The color parameters were measured using a Minolta colorimeter (CR‐400, Minolta, Osaka, Japan). Total volatile basic nitrogen and thiobarbituric acid reactive substances (TBARES) were evaluated using the protocol detailed by Snoussi et al. (2022).

Texture measurements of Merguez samples were performed via a cutting test using a texture analyzer (TVT 6700, Perten, Sweden). The Merguez samples were subjected to cutting using a Bratzler probe. The test and retraction speeds were 0.5, and 1.0 mm/s, respectively and, the obtained results were processed using the TexCalc software. Hence, textural parameters were measured from each strength versus time curve: maximum cutting strength (N), and cutting resistance (positive area under the curve, N·s). Measurements were carried out at room temperature (≈20°C) and with five replicates for each formulation.

#### 2.7.2 Sensory Attributes Analysis

Sensory analysis was evaluated by a hedonic test, which aimed to determine the effect of nanoemulsion preparation on cooked sausage (Merguez) samples. Different characteristics were evaluated like flavor, taste, texture, odor, visual appearance, and overall acceptability, through 7 points in hedonic scales, were 7 extremely like, and 1 extremely dislike by a group constituted of 40 untrained panelists in cooked Merguez samples.

#### 2.7.3 Shelf Life Estimation

The total viable count (TVC) was assessed using the procedure described by AOAC (2002). Briefly, 10 g of Merguez samples were aseptically measured, and mixed with physiological saline for 7 min. The total viable count (TVC) is evaluated by inoculating 0.1 mL proportions after serial 4 at fold dilutions with NaCl solution (0.90%) on plate count agar (PCA) and cultivated at 37°C for 24 h.

The total viable count results of sausage (Merguez), at different storage temperatures (4°C, 15°C, 25°C), were used to estimate the shelf life of Merguez samples. The predicted model, applied in this current study, is the modified Gompertz model described by the following expression (Zwietering et al. 1990), Equation (11):

(11) $$\mathrm{Log}N_{t}=\log N_{0}\log\left(\frac{N_{\max}}{N_{0}}\right)\times \exp\left\{-\exp\left[\frac{2.7182\times \mu_{\max}}{\log\left(\frac{N_{\max}}{N_{0}}\right)}\times \left(\mathrm{Lag}\cdot t\right)+1\right]\right\}$$

With *N*_*t*_ signifying the number of microbial cells at time *t* (CFU/g), *N*_0_ and *N*_max_ represented the initial and maximum number of microbial cells (CFU/g), respectively. *μ*_max_ was the maximum specific growth rate (day^−1^), and Lag was the lag time (days). The effect of the maximum specific growth rate and the lag, as a function of temperature, is given by the following expressions, Equations (12 and 13):

(12) $$\sqrt{\mu_{\max}}=\beta_{\mu }\left(T-T_{\min\mu }\right)$$

(13) $$\sqrt{\frac{1}{\mathrm{Lag}\;t}}=\beta_{\mathrm{lag}}\left(T-T_{\min\mathrm{lag}}\right)$$

where *T* is the temperature (°C), *T*_min *μ*_, and *T*_min lag_ are the extrapolated minimum growth temperatures (°C) for bacterial cells, *β*_*μ*_, and *β*_lag_ are the regression coefficients.

### 2.8 Statistical Analysis

All results were subjected to an ANOVA test using SPSS software (IBM, Corporation, version 21, Chicago, IL, USA), and were expressed as mean ± SD. They compared by Duncan test at *p* < 0.05, which is considered significant. Two statistical parameters were used to validate the modeling of microbiological results, as described by the following expressions (Equations 14 and 15):

(14) $$\text{RMSE}=\sqrt{\frac{\sum_{i-1}^{n}{\left(Y_{\mathrm{ei}}-Y_{\mathrm{pi}}\right)}^{2}}{n}}$$

(15) $$R^{2}=1-\sum_{i=1}^{n}\left(\frac{{\left(Y_{\mathrm{ei}}-Y_{\mathrm{pi}}\right)}^{2}}{{\left(Y_{m}-Y_{\mathrm{pi}}\right)}^{2}}\right)$$

With *n* is the number of experiments whereas *Y*_ei_ and *Y*_pi_ are the experimental and the predicted rheological and microbiological results, respectively. *Y*_*m*_ is the average percentage of the microbiological and rheological results. The accuracy and validation of the model were measured based on the *R* ^2^ and RMSE. In general, the lower the RMSE and the higher the *R* ^2^, the more accurate the model.

## 3 Results and Discussion

### 3.1 Optimization of Capsaicin Encapsulation in Oil–Water Nanoemulsion

#### 3.1.1 Oil Droplet Size Determination

**FIGURE 1:**
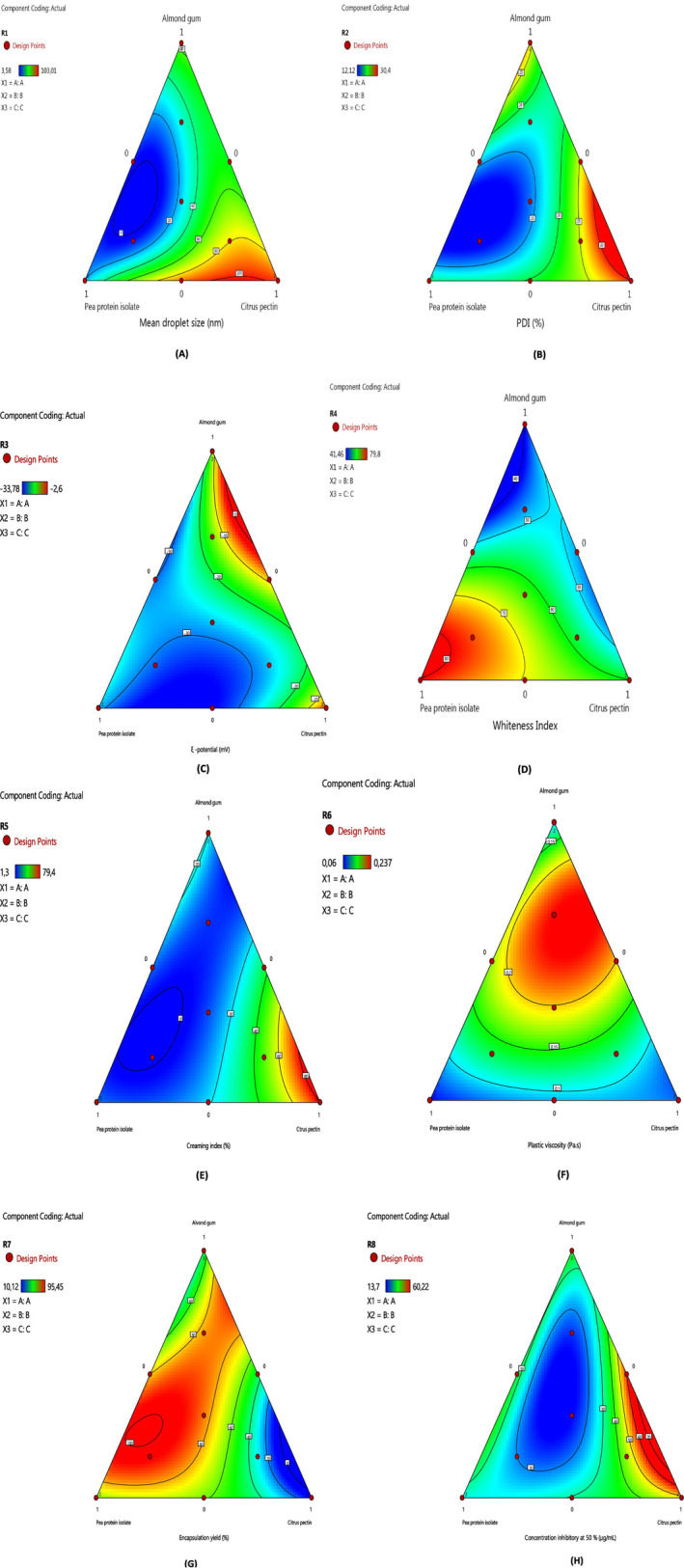
Contour curves for the selected materials on mean droplet size (d[4,3], nm) (A), polydispersity index (PDI) (B), ξ‐potential (mV) (C); whiteness index (D), creaming index (CI, %) (E), plastic viscosity (μ_c_, Pa·s) (F), encapsulation yield (%) (G), and antioxidant activity (IC_50_, μg/mL) (H).

The oil droplet sizes of the formulated nanoemulsions are nanometric forms that varied from 3.58 to 103.01 nm, and the results demonstrated significant differences between formulations (*p* < 0.05). Also, as shown in Table ANOVA (Table 2), the effect of independent variables like pea protein isolate, almond gum, and citrus pectin are significant on mean droplet size with determination, and adjusted regression coefficients of 0.96, and 0.91, respectively. The experimental results were well‐fitted to a special cubic model, as given by the Equation (16) in Table 2. In addition, the results revealed that the citrus pectin was the most significant variable on the increase of mean droplet size (d_[4,3]_), followed by the almond gum, and pea protein isolate (*p* < 0.05). The mean droplet size (d_[4,3]_) of nanoemulsions encapsulated with only pea protein isolate (F3), and citrus pectin (F12) were respectively 63.50 and 103.01 nm, which were significantly higher than the one formulated with almond gum (F1) (20.40 nm) (*p* < 0.05). Furthermore, these results are in accord with those observed by the optical microscope. As indicated in the ANOVA Table, the interaction between almond gum and pea protein represents the most variable for mean droplet size reduction (*p* < 0.05). A small droplet size exhibits a more stable nanoemulsion than the one with a large droplet. It presents a more homogeneous structure and stable systems and avoids creaming, sedimentation, flocculation, coalescence, and gravitational separation due to the sufficient droplet's Brownian motion (Thadros et al. 2004; Oca‐Ávalos et al. 2017). However, nanoemulsions with higher citrus pectin proportions have a large droplet size and exhibited creaming, and phase separation as also found in the work of Dickinson (2019). In addition, nanoemulsion, with 5% almond gum, was more viscous due to higher mass fraction, which also explicates the larger size droplets in F1. Hence, these findings revealed that the type of natural biopolymers significantly affected the mean droplet size (d_[4,3]_) of O/W nanoemulsions, and it increased in the following order: almond gum < pea protein isolate < citrus pectin. The higher value of droplet size of pectin formulation could be related to the lower electrostatic repulsion among droplets caused by the lower adsorption of citrus pectin on the oil–water interface (Guerra‐Rosas et al. 2016). Concerning the pea protein isolate, the higher droplet size explicated the phase separation previously obtained, which could be caused by the depletion flocculation due to an excess in protein isolate. The depletion force in a pea protein isolate‐based nanoemulsion is related to the oil droplet sizes (Radford and Dickinson 2004). Smaller droplets, containing capsaicin, and dispersed in the aqueous phase, form a tighter arrangement, and a lower mobility of droplets, which increase the shear resistance. However, Figure 1A indicated that the droplet size values of the nanoemulsions containing the combination of almond gum, and pea protein isolate were significantly small. Hence, these data demonstrated that the interaction of almond gum, pea protein isolate, and citrus pectin together modify positively the nanoemulsion morphology, and the oil droplet size. This indicated that oil droplets of capsaicin nanoemulsions are covered with surface‐active biopolymers and stabilized efficiently. Furthermore, the incorporation of almond gum into pea protein isolate, and citrus pectin produced smaller droplets and increased their number, which coated the surface area and ameliorated the stability of O/W nanoemulsions. Thus, these results revealed the role of almond gum on the droplet size and structure of nanoemulsion. Our findings are in agreement with those found by Mahfoudhi et al. (2012) who indicated that the increase in almond gum concentrations until 5% (w/w), decreases the mean droplet sizes of emulsions where the almond gum can lid the oil–water interface of the oil droplets, and improve the homogeneity, and emulsion stability. According to Tang et al. (2023), the pea protein, and pectin create hydrophobic interactions, and they form smaller droplets with higher emulsion stability.

**TABLE 2:** Statistical parameters for model prediction describing the relationship between factors and responses.

Response	Predicted model	Expression	*R* ^2^	$R_{\mathrm{adj}}^{2}$	CV (%)
Mean droplet size (nm)	Special cubic	$\text{mean droplet size}\;\left(\mathrm{nm}\right)=65.39\times A+18.06\times B+98.96\times C-162.14\times A\cdot B+125.98\times B\cdot C$ (16)	0.96	0.91	9.80
Polydispersity index	Cubic	$\mathrm{PDI}\;\left(\%\right)=26.35\times A+18.98\times B+29.95\times C-27.61\times A\cdot B-11.66\times A\cdot C-16.60\times B\cdot C-127.14\times A\cdot B\cdot C-$ $16.20\times A\cdot C\cdot \left(A-C\right)$ (17)	0.987	0.962	5.51
ξ‐potential (mV)	Special quartic	$\xi -\text{potential}\;\left(\mathrm{mV}\right)=-12.79\times A-28.72\times B-2.82\times C-72.49\times B\cdot C-963\times A\cdot B\cdot C^{2}$ (18)	0.974	0.93	8.12
Whiteness index	Cubic	$\mathrm{WI}=41.33\times A+79.67\times B+60.17\times C-27.18\times A\cdot B+195.11\times A\cdot B\cdot C-121.26\times A\cdot B\cdot \left(A-B\right)$ (19)	0.994	0.983	2.98
Creaming index (%)	Cubic	$\mathrm{CI}=20.31\times A+10.34\times B+79.61\times C-55.71\times A\cdot C-103.77\times B\cdot C-214.74\times A\cdot C\cdot \left(A-C\right)$ (20)	0.996	0.988	7.30
Plastic viscosity (Pa·s)	Cubic	$\mu_{c}=0.0999\times A+0.0599\times B+0.0799\times C+0.3988\times A\cdot B+0.4383\times A\cdot C+0.0783\times B\cdot C+0.4297\times$ $A\cdot B\cdot C+0.2020\times A\cdot B\cdot \left(A-B\right)+0.6220\times A\cdot C\cdot \left(A-C\right)$ (21)	0.999	0.998	1.62
Encapsulation yield (%)	Cubic	$\text{Encapsulation yield}\;\left(\%\right)=50.36\times A+62.52\times B+10.08\times C+94.76\times A\times B+59.72\times A\times C+100.52\times$ $B\times C+407.71\times A\times B\times C-237.66\times A\times B\times \left(A-B\right)-411.42\times A\times C\times \left(A-C\right)$ (22)	0.999	0.99	0.94
Antioxidant activities (IC_50_, μg/mL)	Cubic	${\mathrm{IC}}_{50}\;\left(\mathrm{\mu g}/\mathrm{mL}\right)=36.30\times A+26.17\times B+47.86\times C+8.95\times A\cdot B+72.85\times A\cdot C-25.77\times B\cdot C-782.71\times$ $A\cdot B\cdot C-52.10\times A\cdot B\cdot \left(A-B\right)-207.44\times A\cdot C\cdot \left(A-C\right)$ (23)	0.999	0.996	0.73
Antimicrobial activities (mm)	a	The model was insignificant at *p* > 0.05	nd	nd	nd

^a^ All experimental results were tested by different models.

Abbreviations: A, almond gum; B, pea protein isolate; C, citrus pectin; CI, creaming index; CV, coefficient of variance; IC_50_, inhibitory concentration at 50%; nd, not determined; PDI, polydispersity index; WI, whiteness index.

#### 3.1.2 Polydispersity Index (PDI)

The polydispersity index (PDI) values of the formulated nanoemulsions are shown in Table 4 and vary from 12.12% to 30.4% depending on almond gum, pea protein isolate, and citrus pectin proportions. Generally, the PDI values range from 0 to 1 indicating monodisperse and polydisperse systems, respectively (Danaei et al. 2018; Snoussi et al. 2022). Indeed, the cubic model was significant at *p* < 0.05 with a higher regression coefficient (*R* ^2^) of 0.9872. Besides, all linear, interaction, and cubic terms were significant on the polydispersity index (*p* < 0.05). As indicated in Figure 1B, the PDI values were higher in the nanoemulsions containing higher pea protein isolate, and almond gum proportions. However, the obtained results demonstrated that formulation with citrus pectin, and almond gum showed the highest PDI values. The difference in PDI can be attributed to the concentration of emulsifiers, which covered all the emulsion droplets, and excess emulsifiers might not be used to cover the interfaces, instead, forming emulsifier layers over the preexisted emulsion droplets, resulting in large‐size droplets and therefore higher values of PDI as indicated by Walia and Chen (2020). Therefore, the blue region is constituted of pea protein isolate, and almond gum proportions are supposed monodisperse indicating a great homogeneity and less aggregation or agglomeration of nanoemulsion. Hence, the obtained data revealed that the cubic model is the best to describe the variation of PDI as a function of the independent variables. In addition, the cubic model of the PDI, predicted by removing the insignificant terms (*p* > 0.05), and expressed in terms of coded variables, is given by the expression 17.

From the predicted Equation (17) in Table 2, it can be seen that all the linear terms were positive and exhibited a negative effect on PDI. While the interaction terms had a good effect on the reduction of PDI values. According to Figure 1B, the variation of the PDI response is shown by the iso‐response curves according to different optimized factors in the experimental field. It is clear that a slight change in the proportion of almond gum could affect the optimal zone of nanoemulsions which explains the important role of this biopolymer on PDI. It can be seen also that the zone delimited by the area in red contains higher proportions of citrus pectin (represents polydispersed nanoemulsions) whereas, the blue zone indicates the optimal phase where the PDI is low (represents the monodispersed nanoemulsions). As mentioned by Telmoudi et al. (2023) the polydispersity index is the cause of partial coalescence. These trends are basically consistent with the changing trend of mean droplet size. Furthermore, it is important to note that almond gum was less efficient than the pea protein isolate, but it performed better than it did in terms of PDI. This could be related to the influence of the emulsifier kinetics of surface adsorption on PDI whereas the small z‐mean diameter is related to the effect of the emulsifier on interfacial tension as aforementioned by Donsì et al. (2012). To conclude, this study indicated that the nanoemulsions are monodisperse by coquette‐cell emulsification, showing that this type of shear is a successful method for formulating stable O/W nanoemulsions.

#### 3.1.3 ξ‐Potential

The ξ‐potential is a tool for concentration determination of the ionic species at droplet's surfaces. The ξ‐potential values of the O/W nanoemulsions, depending on almond gum, pea protein isolate, and citrus pectin proportions, are shown in Figure 1C. Measurements revealed that the ξ ‐potential values varied from −2.60 to −33.78 mV. The highest ξ ‐potential was noticed for the nanoemulsion prepared by higher pea protein isolate, and lower citrus pectin proportions. Likewise, when a higher protein amount was reached, the ξ‐potential became superior to −30 mV, indicating a stronger interparticle electrostatic repulsion existed between pea protein isolate, AL, and CP. This observation is well in concordance to that observed by Zhao et al. (2022). As demonstrated by the ANOVA Table 2, the findings indicated significant differences for nanoemulsions at *p* < 0.05. Therefore, it is clear that the use of biopolymers induced a change of ξ ‐potential in the O/W nanoemulsion and their values were significantly different (*p* < 0.05) depending on the biopolymer type. Thus, the coefficient of correlation (*R* ^2^), and adjusted coefficient of determination ($R_{\mathrm{adj}}^{2}$) were 0.974, and 0.93, respectively, which revealed that the special quartic model is adequate to describe the variation of ξ ‐potential as a function of independent variables (*p* < 0.05) (Table 2). Hence, this model presents a good predictive ability and describes the effect of single biopolymers and their binary and ternary mixtures on the ξ ‐potential response. Similarly, the coefficient of variance (CV) which is an estimation of the reproducibility of the selected model, was less than 10% which proves good reliability and a very high degree of precision of the experimental ξ‐potential values retrieved in this study (Chouaibi et al. 2020). Also, the results of the estimated coefficients of regression indicated that the concentration of pea protein isolate was the most significant than the almond gum and citrus pectin (*p* < 0.05). As indicated in Table 2 the linear terms of the independent variable were significant at *p* < 0.05. In addition, the general cubic model of the ξ ‐potential response, predicted by removing the insignificant terms (*p* > 0.05) and expressed in terms of coded variables, is given by the simplified expression (Equation 18 in Table 2).

From the predicted equation, it can be seen that the single variable terms are negative and exhibited a good effect on ξ ‐potential. The most significant linear variable was noted in the pea protein isolate. Besides, the interaction variable between almond gum and citrus pectin had a significantly negative effect on ξ ‐potential.

Figure 1C shows the results of the mixture contour plots of the predicted model which are represented by the mutual positioning of 13 points in the ABC triangle to study the interaction effects of the biopolymers on ξ ‐potential. The predicted values are represented in a ternary phase curve. The iso‐response curves are hyperbolic branches confirming that there is an interaction between the three independent variables. All nanoemulsions formulated were considered stable as they were relatively higher than −30 mV. The highest ξ ‐potential values are observed in the blue regions corresponding to higher and lower compositions of pea protein isolate and citrus pectin, respectively, indicating that the protein isolate had the highest charges than the almond gum and citrus pectin. According to Figure 1C, the values of ξ ‐potential decreased negatively toward the edge of the almond gum‐pea protein isolate. However, the highest values of ξ ‐potential were placed on the vertex of pectin and almond gum proportions. As can be seen from Figure 1C, the ξ ‐potential values of nanoemulsions increased when the proportions of citrus pectin and almond gum increased. This was because of the acidic character of pectin. Pectin is more stable in acidic environments (Pal and Bera 2020). Furthermore, the addition of more pea protein isolates and almond gum to the mixture increased the ξ ‐potential of the O/W nanoemulsion. This could be attributed to the interactions between the biopolymers. In fact, during encapsulation of active compounds, the interaction of biopolymers such as polysaccharides‐proteins could be influenced by the pH by affecting the performance or structure of the complex system (Cortés‐Morales et al. 2021). It could change the molecular structure, and charge by ensuring the molecules' association, and the electrostatic interactions (Cortés‐Morales et al. 2021). For instance, Ma and Chatterton (2021) reported that the pH can be affected by the electrostatic or hydrophobic interaction between the ionic natural gums and protein at an acidic level (pH = 3–5) to ameliorate emulsion stability but at neutral pH, the interaction does not exist. The interaction between pectin and pea protein isolate is highly dependent on pH in which both polymers give opposite charges at acidic pH and negative charges at higher pH which inhibit the formation of complexes as discussed by Wang et al. (2019). In the same way, Shamsara et al. (2017) reported that the interaction between polysaccharide‐polysaccharide in O/W nanoemulsion could be affected by pH when they studied the electrostatic interaction of apricot gum and pectin complexes and showed that the pH changed during the formulation of nanoemulsions. Our results are in great concordance with those obtained by Sadeghnezhad et al. (2020) who studied the effect of pea protein isolate, pectin, and *Zedo* gum (known bitter almond gum) on the physicochemical properties of bioactive edible film. These findings suggest that stable and uniform nanoemulsions can be successfully formed by pea protein, almond gum, and citrus pectin using a shear‐cell couette.

#### 3.1.4 Whiteness Index

The whiteness index is an indicator of opacity demonstrating a stable nanoemulsion. This parameter is positively correlated to their turbidity values. From the experimental design, the whiteness index values varied from 41.46 to 79.80 depending on the biopolymer's proportions. As indicated in ANOVA Table (Table 2), the cubic model is significant with the coefficient of determination (*R* ^2^) and adjusted coefficient of regression ($R_{\mathrm{adj}}^{2}$) being 0.9943, and 0.9828, respectively, revealing that the cubic model is adequate to describe the variation of whiteness index as a function of independent variables (citrus pectin, pea protein isolate, and almond gum) (*p* < 0.05). Interestingly, the statistical data indicated that the linear terms are the most significant variables (*p* < 0.05). In general, a lower value of WI indicates much transparency of the nanoemulsion. The lower and higher WI values were observed in red and blue regions corresponding to higher almond gum, and pea protein isolate proportions, respectively. The difference in WI is probably dependent on the purity of the used biopolymers. In addition, the cubic model of the WI, predicted by removing the insignificant terms at *p* > 0.05 (Table 2) and expressed in terms of coded variables, is given by the expression (Equation 20 in Table 2).

According to Figure 1D, the iso‐response curves allow us to visualize the variation of the WI response according to the different proportions of almond gum, pea protein isolate, and citrus pectin. It can be observed that the proportion of almond gum could decrease the transparency of nanoemulsions which explains the fundamental role of almond gum on WI. The lightness of gums is related to their concentration of tannin (Bashir et al. 2018). The optimal range in the curve was delimited by the area in red in the graph. Moreover, the results of the ternary curve confirmed that the interactions between the three factors were significant (*p* < 0.05).

#### 3.1.5 Creaming Index Determination

The physical stability of nanoemulsions was assessed by the creaming index (CI) which constitutes a balance among the aqueous and oil phases as described by Liu et al. (2020). It gives information concerning the extent of aggregation as well as the separation of oil droplets in the aqueous phase (Sui et al. 2017). As can be seen from these results, no phase separation was observed in the majority of formulated nanoemulsions that consisted of pea protein isolate, and almond gum, which indicates that they were more stable than the others hence, the creaming index was negligible. The lowest value was found in higher pectin proportions, and the highest value was observed in higher proportions of AL and PPI. As indicated in Table 2, the *R* ^2^ and $R_{\text{adjusted}}^{2}$ were 0.996, and 0.9881, indicating a good relationship between the cubic model and the experimental values of the creaming index (*p* < 0.05). Besides, the linear, and interactions between almond gum, pectin, and pea protein isolate were positively significant (*p* < 0.05). It can be seen that when the amount of pea protein isolate increased, nanoemulsions exhibited more creaming stability. This could be explained by the depletion flocculation because of an excess of biopolymers in the aqueous phase, especially for protein isolate, which caused the non‐adsorbed of protein (Dickinson 2019). Nevertheless, when the protein amount is low, the creaming index increases this is due to smaller droplets having a larger surface area, and requiring more protein amount. Mohammed et al. (2020) found similar results in which *Nigella sativa* oil nanoemulsions prepared with high sodium caseinate concentration caused phase separation and instability during the storage time induced by the depletion flocculation. This mechanism had a large influence on the creaming stability of pea protein isolate, and it could be caused by a flocculated networks compaction or higher concentration of continuous phase, which retarded the formation of the viscous transient droplet network (Liang et al. 2014). In addition, the interaction between almond gum and citrus pectin in nanoemulsions exhibited less CI than the one formulated with only pea protein isolate, which proves that the conjugation of biopolymers with protein isolate affect positively the stability of nanoemulsions. Ma and Chatterton (2021) reported that the addition of biopolymers (natural gums and pectin) with protein augmented the electrostatic or steric repulsion and the adsorbed layer's thickness to enhance emulsion stability against environmental conditions. Furthermore, stable structures of the majority of nanoemulsions are related to the good emulsifying and thickening properties of almond gum and pectin, respectively. Mahfoudhi et al. (2012) reported that the increase in the almond gum concentration decreased the CI and increased the stability of the emulsion which became more homogeneous. Verkempinck et al. (2018) added that citrus pectin is an emulsion stabilizer that enhances the steric, and electrostatic interactions depending on the pectin degree of methyl esterification and pH of the continuous phase in emulsion. The interfacial tension among the water and oil phases can be decreased by citrus pectin, which improves the stability of creaming and emulsions (Leroux et al. 2003). Moreover, emulsion stability depends on many factors such as the particle size, the addition of thickening agents, and the viscosity (Riquelme et al. 2020). Hence, we can conclude that when the almond gum and pea protein isolate were used together, the stability of the emulsion was succeeded. The simplified cubic model of the creaming index was given by the simplified expression (Equation 20 in Table 2).

**FIGURE 2:**
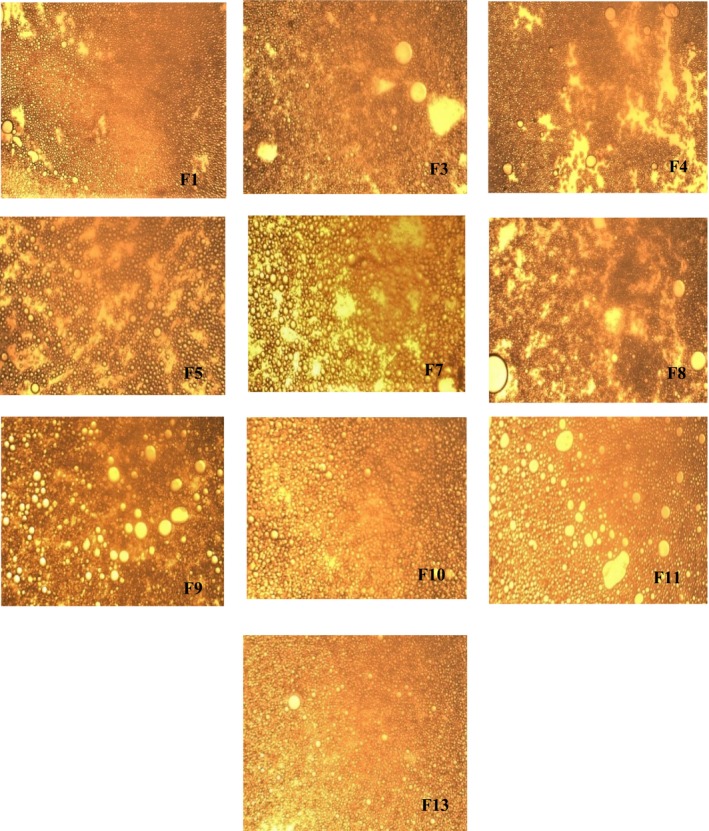
Micrographs of formulated O/W nanoemulsions containing capsaicin stabilized with almond gum, pea protein isolate, and citrus pectin (F_1_ = F_2_; F_3_ = F_6_, and F_4_ = F_12_).

The optical microscope observations of the O/W nanoemulsions encapsulated with the three used biopolymers showed the presence of bright droplets of sunflower oil with spherical shapes dispersed in the aqueous continuous phase, and it was the most common morphology of the produced nanoemulsions (Figure 2). The size and number of oil droplets varied between the samples wherein the majority showed more uniform and wrapped oil droplets hence, it seems that they were monodispersed. Nanoemulsions F5, F10, and F11 showed less uniform droplets and exhibited spaces between the oil droplets while F13 exhibited a more uniform and small droplet size. The small droplet size of formulated nanoemulsions exhibited greater stability for aggregation and following gravitational separation. However, it is clear that the nanoemulsion formulated by citrus pectin showed higher droplets as presented by F4 and F8. During emulsification, the charged macromolecule units disperse into the aqueous solution. Hence, the complex is tightly adsorbed at the oil/water interface, thus providing a steric barrier against aggregation, flocculation, and sedimentation. These obtained observations were well confirmed to the mean droplet size, polydispersity index, and ξ‐potential results mentioned above. Therefore, these findings confirmed the important role of almond gum, and their interaction with pea protein isolate used to improve the stability of O/W nanoemulsion.

#### 3.1.6 Plastic Viscosity

**FIGURE 3:**
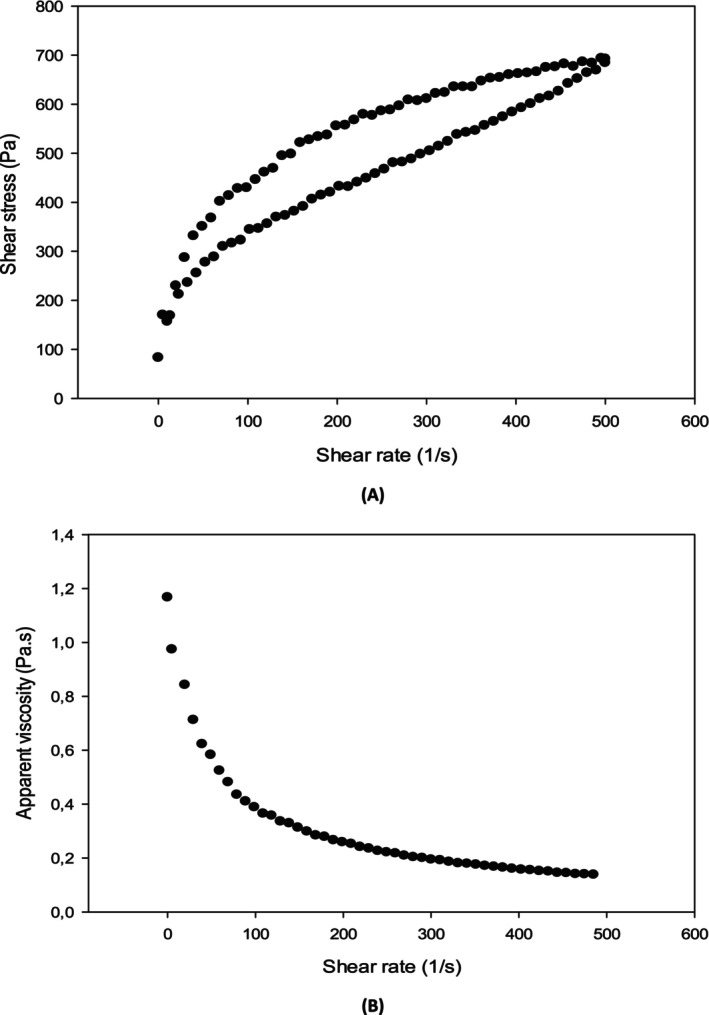
Flow curves (A) shear stress vs. shear rate, (B): Apparent viscosity versus shear rate of O/W nanoemulsion (F10) formulated by 1/3 of AL, 1/3 of PPI, and 1/3 of CP at 20°C.

The flow curves of the formulated nanoemulsions were determined by plotting shear stress as a function of shear rate. The flow curve (Figure 3A) showed a rheogram in the form of hysteresis loops. This property is observed in the food emulsions due to structural breakdown after shear application. The flow curves obtained for each sample were presented in Figure 3B and showed that the apparent viscosity decreased gradually as the shear rate increased, which implied that the shear stress increased with the shear rate. Obviously, we can conclude that the rheological behavior of all formulated nanoemulsions was that of a non‐Newtonian with pseudoplastic (shear thinning) behavior (Figure 3). The pseudoplastic behavior can be explained by a disentanglement of oil droplets and the arrangement of the microstructure of polymers caused by the application of shear stress during the shearing process (Samanta et al. 2010). Kumar and Mandal (2018) found similar behavior in oil‐in‐water nanoemulsions where it enhanced the control of mobility, and they reported that the reduction in apparent viscosity can be related to the Brownian movement of droplets caused by the rupture of nanoemulsion structure. As can be observed in Table 1, F13 samples presenting the conjugation of all biopolymers together showed a higher plastic viscosity which indicates that a more viscous nanoemulsion was produced. It can be also seen that the conjugation of two biopolymers produced then a higher viscosity except for F4 which exhibited the lowest apparent viscosity. Hence, it is clear that the viscosity depends on the amount and type of biopolymers used. The more the viscosity is higher, the more the droplet sedimentation is small, which increases the nanoemulsion stability (Li et al. 2020). The higher viscosity could be related to the interaction between droplets and droplets. It increased when the energy dissipation increased due to the distorted flow field caused by the generation of the dispersed phase in the continuous phase (Ghannam and Selim 2016). In general, the rheological behavior could be affected by the continuous, and dispersed phase viscosity, oil droplet size, temperature, shear rate, stirring, and emulsifiers (Eddine Djemiat et al. 2018).

Mathematical modeling was applied to find the best rheological model that can describe the rheological behavior of nanoemulsions using Casson and Power models. Based on the statistical parameters, the Casson model was the most suitable for describing the behavior of the prepared nanoemulsions. The estimated parameters of models should not be negative (Koocheki et al. 2013). The rheological parameters showed that the coefficient of determination (*R* ^2^) values varied between 0.721 and 0.924. On the contrary, the values of the plastic viscosity varied from 0.06 to 0.237 Pa·s, which were significantly different (*p* < 0.05). The highest plastic viscosity was found in the formulation stabilized by the three biopolymers with a higher concentration of almond gum. Consequently, we can conclude that the almond gum has a fundamental role in increasing the apparent viscosity as found by Rezaei et al. (2016) who reported that the viscosity increased by increasing the almond gum concentration where a shear thinning behavior was obtained. Panchev et al. (2009) found the same behavior for pectin nanoemulsion but the Herschel–Bulkley law was more adequate to describe its behavior. The conjugation of biopolymers such as gums and pectin with pea protein isolate and without complexation increased the continuous phase viscosity which decreased the mobility, aggregation, and coalescence of oil droplets and enhanced the emulsion stability (Ma and Chatterton 2021).

The plastic viscosity values (*μ*_*c*_) were determined in all formulations based on experimental design and the obtained results revealed that the cubic model is significant with *R* ^2^ and $R_{\text{adjusted}}^{2}$ are 0.999, and 0.998, respectively. The CV and lack of fit indicating the cubic model can be used to describe the experimental design. Also, all linear, and interaction terms are significant (*p* < 0.05). The most linear and the interaction variables were almond gum, and interactions between almond gum and citrus pectin, respectively. Besides, the lowest values of plastic viscosity can be attributed to the reduction of droplet size. These findings are in concordance with the study of Salvia‐Trujillo et al. (2015). The predicted cubic model is given by the expression (Equation 21 in Table 2).

#### 3.1.7 Encapsulation Yield of Capsaicin

The encapsulation yield represents the important chemical parameters that affect the nanoemulsion stability. There are many factors, which affect the encapsulation yield like biopolymer type, encapsulation methods, and storage methods of nanoemulsions (Chouaibi, Mejri, et al. 2019; Chouaibi, Rezig, et al. 2019; Snoussi et al. 2022; Telmoudi et al. 2023). ANOVA Table 2 showed that the cubic model presents a significant difference between the response and factors (*p* < 0.05). As indicated in the ANOVA Table, the special cubic is significant with adjusted and determination regressions of 0.999, and 0.99, respectively. Although, all linear, interactions between pea protein isolate, and citrus pectin are significant on encapsulation yield (*p* < 0.05). Interestingly, the pea protein isolate is the most significant independent variable on the encapsulation yield of capsaicin using the nanoemulsification process (*p* < 0.05). However, the obtained results indicated that the pea protein isolate increased the encapsulation yield of capsaicin. This is due to the interaction between capsaicin, and protein isolate to form a complex product. In contrast, the encapsulation yield increased is probably related to protein‐polysaccharide that decreased the interfacial tension between the oil, and the aqueous phase. Besides, the increase in capsaicin yield is explained by the interaction between PPI and capsaicin through hydrophobic interaction, so the loading capacity was significantly increased. As indicated in Figure 1G, the highest encapsulation yield of capsaicin was detected in the red region corresponding to higher and lower pea protein isolate, and almond gum proportions, respectively. Hence, the predicted cubic model was expressed by the expression (Equation 22 in Table 2).

### 3.2 Antioxidant Activities by DPPH Test

To compare the antioxidant potential of the formulated nanoemulsions, DPPH radical scavenging activities, were evaluated. Inhibitory concentration at 50% (IC_50_) values of formulated nanoemulsions through DPPH scavenging activity ranged from 13.7 to 60.22 μg/mL. Note that a higher IC_50_ value indicates a lower antioxidant activity of the nanoemulsions. The highest antioxidant capacity was observed in F10 (13.07 μg/mL) with containing 1/3 of each biopolymer. The conjugation process of almond gum, pea protein isolate, and citrus pectin with equal amounts could protect nanoemulsion against lipid oxidation. Furthermore, it is clear that F11 and F13 exhibited important antioxidant capacities as compared to the other samples while F4 and F8 revealed the lowest values. These results exhibited that the nanoemulsions containing an amount of almond gum and pea protein isolate exhibited higher antioxidant activities. So, these nanoemulsions could be used in the formulation of new food products to increase oxidative stability. This finding is similar to the achieved by Bouaziz et al. (2017) who reported that the almond gum is rich in polyphenols and volatile compounds which showed potential antioxidant activities. This result is in line also with Bashir et al. (2018) who found that almond gum exhibited maximum DPPH˙ inhibition compared to the apricot gum and gum Arabic at the concentration of 1 mg/mL. Samrot et al. (2018) reported that the antioxidant activity increased by increasing the concentration of almond gum polysaccharide. Hence, the antioxidant activities found in all nanoemulsions could be also related to the capsaicin. Akbas et al. (2019) formulated an oleoresin capsicum nanoemulsion stabilized with sucrose monopalmitate and lecithin. They found that encapsulating capsaicin in nanoemulsion enhanced its antioxidant activity in food products that contain high amounts of water. They added that capsaicin nanoemulsions provide antioxidant capacities that can be used to control lipid oxidation. The most responsible compound of this antioxidant activity is the phenolic OH group found in capsaicin, as aforementioned by Okada et al. (2010).

Furthermore, the ANOVA Table that the cubic model presents a significant difference between the response and factors (*p* > 0.05), with higher regression and adjusted coefficients, 0.999, and 0.996, respectively. As indicated in Figure 1H, the lowest IC_50_ was observed in the middle regions of the ternary curve corresponding to high proportions of pea protein and low concentrations of AL and CP. The predicted model of IC_50_ as a function of independent variables was described by the equation (Equation 23 in Table 2).

#### 3.2.1 Antibacterial Activities

**FIGURE 4:**
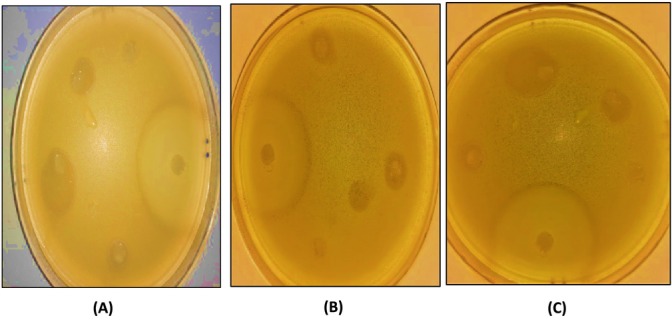
Zone of inhibition of antibacterial activities of some formulated nanoemulsions containing 200 mg/kg of capsaicin. (A) Zone of inhibition of *Listeria monocytogenes* in formulation F5 (2.5% of almond gum + 0.75% of pea protein); (B) Zone of inhibition of *Staphylococcus aureus* in formulation F13 (0.83% of almond gum + 1% of pea protein + 0.167% of citrus pectin); (C) Zone of inhibition of *Escherichia coli* in formulation F13 (0.83% of almond gum + 1% of pea protein + 0.167% of citrus pectin).

The antibacterial activities of formulated nanoemulsions were evaluated by the Agar Diffusion Method, and the results are shown in Table 3. All the nanoemulsions showed the appearance of a clear halo indicating that they possessed antibacterial activities. Figure 4 revealed some examples of nanoemulsions with inhibitory activities on bacteria. Indeed, the inhibition zone varied between 5 and 14 mm where it was moderately large for both Gram‐positive and Gram‐negative bacteria. Besides, sensitivity was also determined, and the obtained results demonstrated that Gram‐positive bacteria were more sensitive to the formulated nanoemulsions (Table 3). This could be related to the more complex structure of the cell wall of Gram‐negative bacteria than Gram‐positive bacteria, where it is dotted with an outer membrane which constitutes a strong barrier (Nikaido 2003). These findings are similar to those found by Hasssanzadeh et al. (2018), who investigated that formulation of garlic oil‐in‐water nanoemulsion were more sensitive to Gram‐positive bacteria ( *Staphylococcus aureus* ) than Gram‐negative bacteria ( *Escherichia coli* ).

**TABLE 3:** Growth inhibition zone (mm) of formulated nanoemulsions based on Agar Diffusion Method.

Formulations	Inhibition zone in disc diffusion assay (mm)				
	*Staphylococcus aureus*	*Listeria monocytogenes*	*Escherichia coli*	*Salmonella arizone*	*Pseudomonas aeruginosa*
F1	7 (+)	8 (++)	7 (+)	8 (++)	7 (+)
F2	6 (+)	8 (++)	6 (+)	7 (+)	8 (++)
F3	11 (++)	7 (+)	9 (++)	9 (++)	12 (++)
F4	10 (++)	6 (+)	11 (++)	11 (++)	9 (++)
F5	9 (++)	13 (++)	9 (++)	10 (++)	8 (++)
F6	9 (++)	13 (++)	10 (++)	11 (++)	10 (++)
F7	8 (++)	6 (+)	6 (+)	8 (++)	9 (++)
F8	6 (+)	7 (+)	6 (+)	5 (−)	7 (+)
F9	13 (++)	10 (++)	11 (++)	11 (++)	12 (++)
F10	8 (++)	6 (+)	9 (++)	10 (++)	7 (+)
F11	6 (+)	8 (++)	6 (+)	6 (+)	7 (+)
F12	9 (++)	11 (++)	10 (++)	14 (++)	10 (++)
F13	13 (++)	7 (+)	11 (++)	11 (++)	8 (++)

*Note:* (−): negative reaction, surface of inhibiotion < 100 mm^2^; (+): Surface of inhibition zone < 200 mm^2^; (++): surface of inhibition zone between 200 and 400 mm^2^.

Moreover, all nanoemulsions displayed a greater antimicrobial activities on all the bacteria with inhibition diameters ranging from 5 to 14 mm. However, the obtained data revealed that the highest inhibition diameters were observed in Gram‐positive bacteria. Specifically, the effect of F9 and F13 against *Staphylococcus aureus, Escherichia coli, Salmonella arizone* and, *Pseudomonas aeruginosa*. However, F5 and F6 presented higher inhibition diameters against *Listeria monocytogenes* . It can be seen that the effect of pea protein isolate, and almond gum was greater than the citrus pectin. Furthermore, an increase in the amount of each biopolymer results in an enlargement of the inhibition zone. Hence, it can be noted that the obtained antibacterial activities are caused by the bactericidal properties of biopolymers used, and probably by the sustained release of capsaicin. Almond gum has antimicrobial effects (Hussain and Jaisankar 2017). Their compounds inhibit the growth of bacteria (Bouaziz et al. 2017). On the contrary, the addition of pectin enhances antibacterial stability as reported by (Cheng et al. 2020). Moreover, pea protein isolate also has antimicrobial activities (Xue et al. 2015). The antibacterial activity can be influenced by many factors such as the nanoemulsion process (Ozogul et al. 2020), the type of emulsifier (Prakash et al. 2017), and the capsaicin concentration (El‐Naggar et al. 2020). As indicated by Lima et al. (2021), the enhancement of the antibacterial activities could be justified by the increase of surface area from smaller droplets permitting passive transport through the outer cell membrane.

While the selected models of inhibition zone for all selected bacteria were insignificantly at *p* > 0.05. Therefore, the results obtained showed that the formulated nanoemulsions can be used as a functional mixture with appropriate antimicrobial activities.

### 3.3 Model Validation

To validate the obtained optimal conditions, an experimental with optimal almond gum, pea protein isolate, and citrus pectin proportions were assessed in four replicates: Almond gum (16.13%), pea protein isolate (73.45%), and citrus pectin (10.42%). The predicted, and experimental responses were statistically compared using the Student test and the results showed no significant variation between experimental and predicted values of mean droplet size (3.58, and 3.80 nm), polydispersity index (12.13%, and 14.06%), ξ ‐potential (−30.53, and −31.20 mV), whiteness index (78.65, and 80.47), plastic viscosity (0.12, and 0.11 Pa·s), creaming index (1.96%, and 2.05%), encapsulation yield (94.06%, and 96.12%), the inhibitory concentration of 50%, antibacterial activities (23.01, and 19.80 μg/mL), respectively (*p* > 0.05). Besides, the obtained data revealed that global desirability was 95.56% indicating the adequacy of the selected mixture design for capsaicin encapsulation using an emulsification process.

### 3.4 Preservation of Sausage (Merguez)

#### 3.4.1 Changes in pH and Color

**FIGURE 5:**
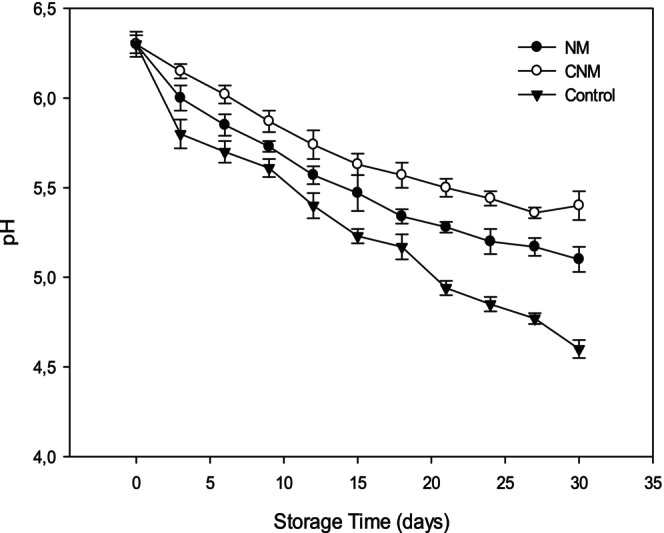
pH variations of the control, and treated Merguez samples during 30 days of refrigerated storage (4°C). Error bars in figures represent standard deviation.

The pH is considered an essential tool for the quality determination of freshness‐meat products. The pH of three samples (capsaicin‐nanoemulsion Merguez (CNM), nanoemulsions‐Merguez (NM), and control (C)) is shown in Figure 5. Similarly, these results indicated that the initial pH value (0 day) was 6.30. In addition, these findings demonstrated that pH values of all Merguez samples significantly decreased with the increase of storage period (*p* < 0.05). However, the pH of sausages with capsaicin nanoemulsion (CNM) decreased slowly and remained insignificant after 20 storage days (*p* > 0.05). The treatment of capsaicin nanoemulsion did not change pH Merguez due to the antimicrobial activities of the capsaicin nanoemulsion. As indicated by Ghribi et al. (2018) and Feng et al. (2020) the decrease in pH values was due to the accumulation of alkaline compounds like ammonia or trimethylamine, and acid compounds from possible microbial spoilage or the degradation of protein and amino acids. At the end of storage, the pH values of control, nanoemulsion‐Merguez, and capsaicin‐nanoemulsion Merguez were 4.60, 5.10, and 5.4, respectively. According to Triki et al. (2013a, 2013b), the pH decrease in Merguez is due to the lactic acid production by *Lactobacillu*s which is often associated with fresh meat products. Thus, the obtained data revealed that the encapsulated capsaicin can reduce the decrease of Merguez pH. In addition, the slow pH decrease of capsaicin‐nanoemulsion Merguez (CNM) can be explained by the synergy effects of pea protein isolate, capsaicin, and other biological substances as described by Li et al. (2024).

**FIGURE 6:**
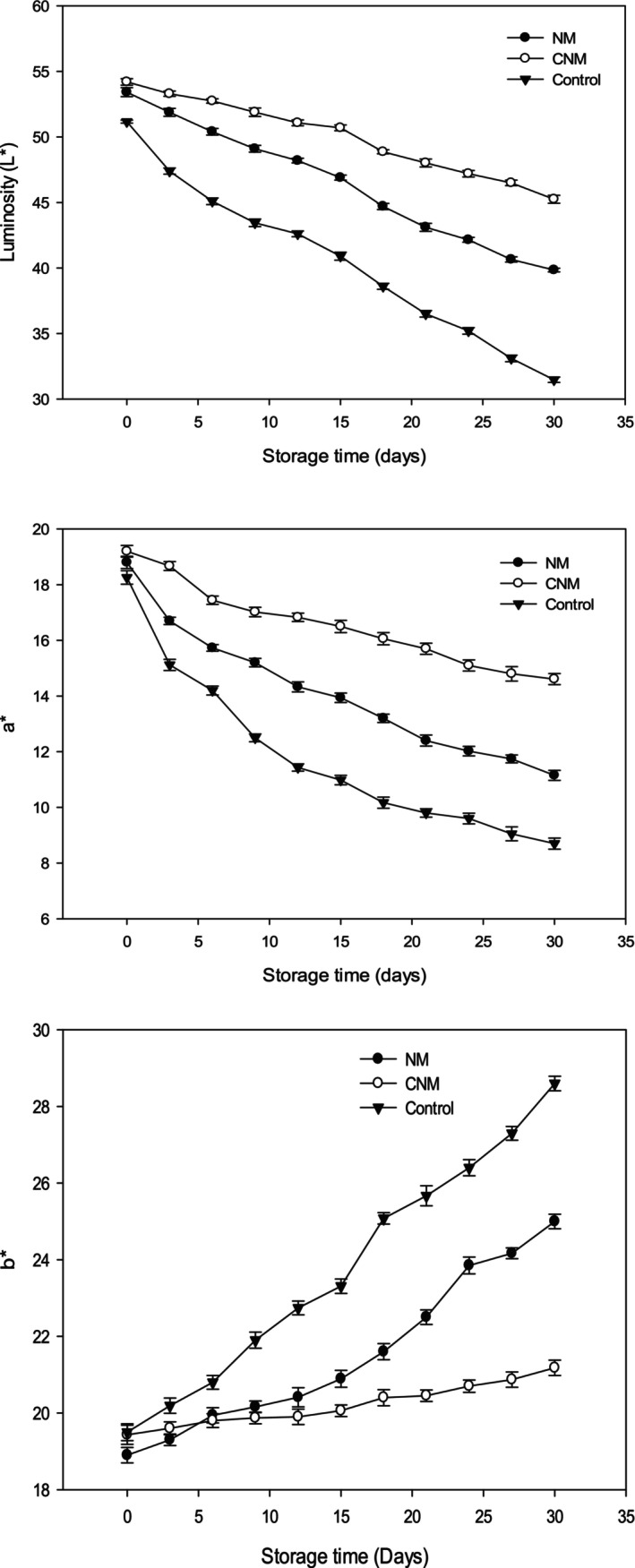
Color parameters (L*, a*, and b*) of the control, and treated Merguez samples with O/W nanoemulsions (NM and CNM), stored at 4°C, and during 30 days of storage time (Error bars in figures represent standard deviation).

The color of meat is an indicator of the freshness of meat products. As can be seen from Figure 6, that color parameters (L*, a*, b*) were influenced by storage period, and treatment with O/W nanoemulsions (*p* < 0.05). Furthermore, the luminosity (L*) of the treated, and control samples decreased with the increase of storage period at 4°C. Indeed, the lowest L* values were observed in the control followed by nanoemulsion‐Merguez (NM), and capsaicin‐nanoemulsion (CNM) Merguez samples. Therefore, these results were in concordance with those found by Triki et al. (2013a, 2013b), and Snoussi et al. (2022) who indicated that the luminosity (L*) of meat products decreased with the increase of storage time. However, all samples showed an increase in b* parameter during storage time. There is no significant variation of the b* parameter of CNM, and NM samples during the first week of storage time (*p* > 0.05). However, these findings revealed that control samples demonstrated the highest b* parameter. These obtained data are similar to those found by Triki et al. (2013a, 2013b) who mentioned that the b* parameter of Merguez increased with the increase of storage time, and they related to the lipid oxidation of Merguez samples. However, the red parameter (a*) of all formulated Merguez showed a decrease during the cold storage time. Interestingly, there is a significant difference between the control, and treated samples by O/W nanoemulsions in terms of the redness parameter (a*) (*p* < 0.05). According to Feng et al. (2020), the decrease in the redness parameter is due to the transformation of red‐colored myoglobin into brown‐colored metmyoglobin. Besides, as reported by Shange et al. (2019), the loss of meat redness is probably explained by lipid oxidation, myoglobin amount, and preservation methods. From these data, it should be concluded that encapsulated capsaicin is as much a natural potential preservative of Merguez.

#### 3.4.2 Physicochemical Characteristics

The water loss presented the most significant physical parameter that affect the final meat quality. As indicated in Table 4, the loss water of all Merguez samples increased significantly with the increase of storage time at 4°C. While the lowest and highest values were detected in CNM, and control samples, respectively. However, there is a significant difference between samples in terms of water loss. The diminution of water loss in the Merguez samples could be attributed to the reduction of protein oxidation and denaturation. Nevertheless, water loss may enhance the loss of protein and lipids because of increased oxidation. These obtained results were similar to those found in Tunisian Merguez elaborated by Triki et al. (2013a, 2013b).

**TABLE 4:** Variation of water loss, and textural parameters of Merguez samples (control and treated samples) during 4°C storage of 30 days.

(Days)	Sample	Water loss (%)	TVB‐N (mg/100 g)	TBARES (mg MDA/kg)	Cutting strength (N)	Cutting resistance (N·s)
0	NM	0	6.55 ± 0.10a	0.17 ± 0.01c	12.14 ± 0.20b	70.19 ± 4.24b
	CNM	0	6.41 ± 0.13b	0.17 ± 0.02f	12.30 ± 0.17a	72.10 ± 4.30a
	C	0	6.56 ± 0.10b	0.17 ± 0.01f	12.40 ± 0.11a	71.80 ± 3.40a
3	NM	5.23 ± 0.10b	6.68 ± 0.09b	0.19 ± 0.01a	11.61 ± 0.21d	64.10 ± 4.70d
	CNM	3.10 ± 0.17b	6.50 ± 0.10a	0.18 ± 0.02e	11.90 ± 0.15c	68.51 ± 3.86c
	C	7.12 ± 0.11a	6.90 ± 0.11b	0.20 ± 0.01f	11.32 ± 0.21a	62.17 ± 4.11a
6	NM	8.30 ± 0.13c	6.84 ± 0.10c	0.22 ± 0.01b	11.10 ± 0.15e	58.32 ± 3.62e
	CNM	4.61 ± 0.12c	6.67 ± 0.06c	0.19 ± 0.02d	11.60 ± 0.14d	64.12 ± 3.53e
	C	10.58 ± 0.18a	7.10 ± 0.14b	0.35 ± 0.02f	10.50 ± 0.13a	53.64 ± 3.22a
9	NM	10.80 ± 0.16d	7.07 ± 0.08d	0.30 ± 0.02a	10.83 ± 0.10d	54.72 ± 3.19f
	CNM	5.31 ± 0.10d	6.89 ± 0.10d	0.24 ± 0.02c	11.07 ± 0.16e	62.12 ± 2.82f
	C	13.90 ± 0.19a	7.30 ± 0.14b	0.60 ± 0.02f	10.06 ± 0.18a	47.17 ± 3.05a
12	NM	12.25 ± 0.15d	7.32 ± 0.05e	0.36 ± 0.03a	10.54 ± 0.20c	51.60 ± 3.44c
	CNM	6.70 ± 0.17e	6.97 ± 0.07e	0.24 ± 0.01b	10.82 ± 0.23e	60.82 ± 2.66d
	C	16.04 ± 0.16a	7.80 ± 0.12b	0.90 ± 0.02f	9.80 ± 0.16 a	43.20 ± 3.10a
15	NM	13.42 ± 0.07e	7.70 ± 0.09f	0.47 ± 0.03ab	11.17 ± 0.03a	54.31 ± 3.74a
	CNM	8.65 ± 0.15e	7.14 ± 0.05f	0.31 ± 0.01a	11.62 ± 0.15b	64.01 ± 3.41b
	C	19.22 ± 0.14a	8.40 ± 0.13b	1.10 ± 0.03f	9.88 ± 0.19a	45.20 ± 3.63a
18	NM	15.82 ± 0.12a	7.98 ± 0.08a	0.53 ± 0.04c	11.63 ± 0.20b	58.11 ± 3.05b
	CNM	10.09 ± 0.14a	7.43 ± 0.08b	0.37 ± 0.03f	12.20 ± 0.11a	70.83 ± 4.01a
	C	22.60 ± 0.12a	10.55 ± 0.10b	1.24 ± 0.02f	9.44 ± 0.14a	47.27 ± 3.19a
21	NM	18.56 ± 0.13b	8.41 ± 0.12b	0.60 ± 0.03a	11.90 ± 0.16d	61.14 ± 3.42d
	CNM	12.07 ± 0.12b	7.83 ± 0.10a	0.46 ± 0.02e	12.80 ± 0.11c	76.06 ± 2.88c
	C	25.19 ± 0.08a	12.03 ± 0.14b	1.30 ± 0.04f	9.84 ± 0.11a	51.07 ± 3.53a
24	NM	20.10 ± 0.18a	9.19 ± 0.07a	0.66 ± 0.05c	12.09 ± 0.19b	65.20 ± 3.15b
	CNM	14.42 ± 0.09a	8.04 ± 0.07b	0.48 ± 0.03f	13.40 ± 0.11a	81.19 ± 4.11a
	C	26.79 ± 0.12a	14.55 ± 0.06b	1.48 ± 0.04f	10.06 ± 0.11a	55.09 ± 3.32a
27	NM	22.66 ± 0.11b	10.21 ± 0.04b	0.74 ± 0.06a	12.20 ± 0.18d	68.39 ± 3.60d
	CNM	16.12 ± 0.08b	8.58 ± 0.05a	0.50 ± 0.04e	13.77 ± 0.20c	86.47 ± 4.18c
	C	29.91 ± 0.12a	15.20 ± 0.06b	1.68 ± 0.05f	10.14 ± 0.11a	57.12 ± 3.40a
30	NM	24.53 ± 0.08b	11.32 ± 0.12b	0.83 ± 0.04a	12.36 ± 0.24d	72.90 ± 4.14d
	CNM	18.40 ± 0.10b	8.94 ± 0.11a	0.57 ± 0.03e	14.08 ± 0.21c	89.20 ± 3.22c
	C	32.70 ± 0.12a	15.88 ± 0.09b	1.80 ± 0.05f	10.35 ± 0.13a	60.11 ± 3.26a

*Note:* Values are mean ± SD of three replicates. Different letters within the same row indicate significant differences in terms of storage time (one‐way ANOVA and Duncan test, *p* < 0.05).

Abbreviations: C, control; CNM, capsaicin‐nanoemulsion Merguez; NM, nanoemulsion‐Merguez; TBARES, thiobarbituric acid reactive substances; TVB‐N, total volatile basic nitrogen.

Total volatile basic nitrogen was applied as a tool for exploring protein oxidation and degradation. The total volatile basic nitrogen (TVB‐N) of three samples (control, and treated with nanoemulsion) is demonstrated in Table 4. From the obtained data, the total volatile basic nitrogen (TVB‐N) increased in all samples over the storage period. The initial value of TVB‐N was 6.85 mg/100 g of samples (CNM, NM, and control). In addition, the results indicated that the variation of TVB‐N varied linearly with storage time. The TVB‐N of CNM, NM, control ranged from 6.41 to 8.94 mg/100 g, from 6.55 to 11.32 mg/100 g, and from 6.56 to 15.88 mg/100 g, respectively. From these obtained results, it is clear that capsaicin can reduce the TVB‐N amount, delay protein decomposition, and lipid oxidation, and therefore prolong the shelf life of Merguez products is over 30 days compared to the control sample. The reduction in TVB‐N of treated samples can be related to the antibacterial activities of capsaicin, and its combination properties and synergistic effects. These obtained findings are in great agreement with those worked by Xin et al. (2023), Safari et al. (2023), and Amiri et al. (2024).

TBARS is a means to evaluate lipid oxidation, and it is negatively related to meat color. As can be seen from Table 4, the TBARS values of all samples increased with the increase of storage time at 4°C. There are significant differences between treated and control Merguez (*p* < 0.05). Hence, the initial TBARS values of CNM, NM, and control are 0.17 MDA mg/kg. At the end of storage time, the TBARS values of CNM, NM, and control became 0.57, 0.83, and 1.8 MDA mg/kg, respectively. These obtained values were comparable to those found by Xin et al. (2023) but they are lower than those found by Snoussi et al. (2022) who prepared thyme essential oil nanoemulsion used for the beef meat preservation. To conclude, the encapsulation of capsaicin in O/W nanoemulsion effectively reduces the oxidation rate of peroxides, lipid hydroperoxides, and secondary products. Indeed, the oxidation delay is due to the barrier formation constituted of capsaicin nanoemulsion at the Merguez surface. Another reason is the synergic effect of capsaicin, and pea protein isolate which the antioxidant activities of complexes are improved, and therefore, the oxidation rate of lipids is reduced.

The texture is an important parameter for consumer's preferences for food products. Two textural parameters such as cutting strength, and cutting resistance were evaluated using a cutting test, and the obtained results were summarized in Table 4. Cutting strength is a textural parameter indicating the firmness of all sausage samples, which significantly decreased sharply during 12 days of storage time. After 15 days of storage, the strength cutting increased significantly from 54.20 to 72.90 N, from 64.24 to 89.20 N, and from 45.11 to 60.02 N of CN, CNM, and control, respectively (*p* < 0.05). However, for the treated samples by O/W nanomeulsions, the results indicated that the cutting strength decreased during the two first weeks of storage time, and increased for the rest of the storage time. Interestingly, the highest cutting strength is detected for CNM and represents a significant difference between the prepared samples (control, and treated samples) (*p* < 0.05). At the end of the storage period (30 days), the shearing strength values of control, NM, and CNM were 10.35, 12.36, and 14.12 N, respectively. Another textural parameter like cutting resistance which indicated the amount of energy required to cut totally the sausage Merguez, was also examined, and the obtained findings revealed that the cutting resistance of CNM, NM, and control ranged from 83.72 to 74.09 N·s, and from 81.87 to 61.10 N·s, and from 80.43 to 48.22 N·s, respectively. Remarkably, the lowest value in cutting resistance was noted in the control followed by NM, and CNM, respectively. Indeed, the decrease in cutting resistance can be related to lipid oxidation, water loss, and protein degradation of sausages. To conclude, it is evident that the capsaicin nanoemulsions improve the textural profile of Merguez, and they are related to the meat protein oxidation, and degradation.

#### 3.4.3 Sensory Attributes Evaluation

**FIGURE 7:**
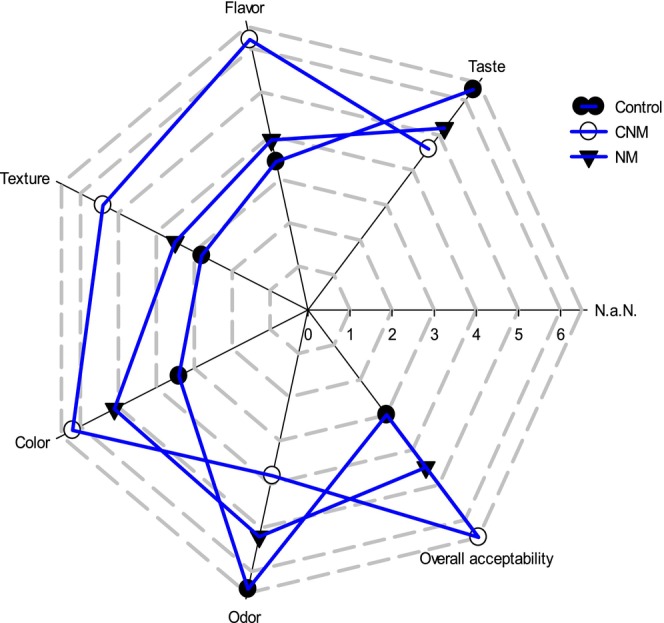
Sensory attributes evaluation of control, and treated Merguez sausages (NM, and CNM) with O/W nanoemulsion at 3 days of storage at 4°C.

The statistical analysis showed that most of the sensory attributes perceived that the three formulated samples (Control, NM, and CNM) were different in terms of taste, odor, flavor, visual appearance, texture, and overall acceptability (Figure 7). Indeed, the data obtained demonstrated that there are significant differences, especially in texture, flavor, odor, visual appearance, and overall acceptability of all Merguez samples (*p* < 0.05). However, it is clear that the highest values of texture, flavor, and overall acceptability were observed in capcaisin‐nanoemulsion Merguez, followed by these prepared only by nanoemulsion. Besides, the control sample showed the highest value in taste and odor. This is explained by the degradation, and oxidation of meat lipids, and proteins of Merguez. These sensorial parameters were positively correlated to the color parameters, textural characteristics, and physicochemical proprieties of Merguez. This indicates that the higher the a* parameter the merguez has, the higher the scores of the color descriptor and the higher overall acceptability are.

#### 3.4.4 Prolongation of Shelf Life

**FIGURE 8:**
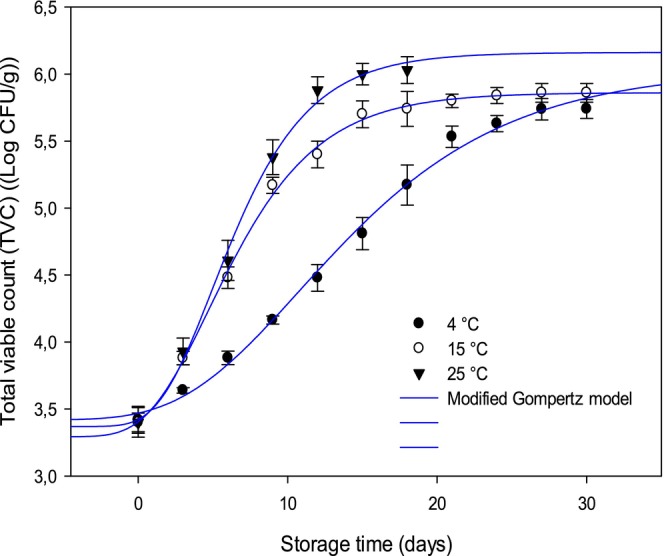
Comparison between experimental kinetic curves of total viable count (TVC) and modified Gompertez model, in capsaicin‐nanoemulsion Merguez samples, stored at 4°C, 15°C, and 25°C (Error bars in figures represent standard deviation).

The shelf life of sausage (Merguez) is determined based on the microbiological results of total viable count (TVC) evaluated at three stored temperatures (4°C, 15°C, and 25°C). The obtained results for TVC are listed in Figure 8. However, the TVC of all Merguez samples increased with the rise in storage time and temperature. The variation of TVC of Merguez was modeled using the modified Gompertz model, and the obtained data of kinetic parameters as well as the statistical parameters, are summarized in Table 5. The results revealed that the initial number of microbial cells (*N*_0_) is not significant in terms of Merguez samples, and storage temperature (*p* > 0.05). However, the maximum of the microbial cells (*N*_max_) and lag time (lag) decreases significantly with the increase in storage temperature (*p* < 0.05). Likewise, the maximum cell growth rate (*μ*_max_) increased with the increase in storage temperature, and it is dependent on the type of treatment of sausage (Merguez). As mentioned by Triki et al. (2013a), the limit level of 6 log CFU/g is the acceptable total microbial quality of standard Merguez sausages. The treatment by capsaicin‐naoemulsion increased significantly the maximum growth rate. Interestingly, there are significant differences between Merguez samples, and the highest maximum cell growth rate (*μ*_max_) was observed in the control followed by NM, and CNM.

**TABLE 5:** Estimated growth parameters of total viable count (TVC) of control and treated Merguez (NM, and CNM) samples based on the modified Gompertz model.

Samples	Temperature (°C)	*N*_0_ (Log CFU/g)	*N*_max_ (Log CFU/g)	*μ*_max_ (days^−1^)	Lag (days)	*R* ^2^	RMSE
NM	4	3.32 ± 0.05	6.80 ± 0.10	0.103 ± 0.001	3.29 ± 0.07	0.998	0.03
	15	3.35 ± 0.04	6.93 ± 0.08	0.43 ± 0.001	1.72 ± 0.10	0.993	0.06
	25	3.36 ± 0.03	7.02 ± 0.12	0.90 ± 0.002	1.10 ± 0.06	0.987	0.02
CNM	4	3.33 ± 0.02	5.73 ± 0.09	0.058 ± 0.001	3.456 ± 0.11	0.996	0.05
	15	3.25 ± 0.05	5.86 ± 0.13	0.245 ± 0.002	2.361 ± 0.08	0.989	0.03
	25	3.30 ± 0.04	6.03 ± 0.10	0.525 ± 0.002	1.922 ± 0.05	0.974	0.03
Control	4	3.40 ± 0.06	7.02 ± 0.07	0.183 ± 0.001	1.863 ± 0.10	0.985	0.06
	15	3.39 ± 0.07	7.31 ± 0.11	0.632 ± 0.001	0.794 ± 0.07	0.998	0.04
	25	3.36 ± 0.05	7.72 ± 0.12	1.36 ± 0.002	0.213 ± 0.09	0.999	0.03

Based on determination coefficients (*R* ^2^) and root mean square error (RMSE), the two primary models were predicted as a function of storage temperature. The combination between the primary models, and these of modified Gompertz was given by the following expression of the microbial growth rate model for CNM stored at 4°C–25°C as below (Equation 24):

(24) $$\log N_{t}=\log N_{0}\left(6.20-\log N_{0}\right)\times \exp\left\{\exp\left[\frac{{\left(0.023T+0.15\right)}^{2}\times 2.7182}{6.20-\log N_{0}}\times \left(\frac{1}{{\left(0.0088\;T+0.508\right)}^{2}}-t\right)+1\right]\right\}$$

where *t* is the shelf life (SL) of Merguez products, and *T* is the selected storage temperature of the meat product (°C). Hence, the predicted shelf life (*t*) of Merguez stored at 4°C–25°C, can be estimated as (Equations (25), (26), (27)):

(25) $${\mathrm{SL}}_{\mathrm{NM}}\;\left(\text{days}\right)=\frac{1}{{\left(0.0192\;T+0.474\right)}^{2}}-\left\{\ln\left[-\ln\frac{\log N_{t}-\log N_{0}}{6.80-\log N_{0}}\right]-1\right\}\times \frac{6.80-\log N_{0}}{{\left(0.03\;T+0.17\right)}^{2}\times 2.7182}$$

(26) $${\mathrm{SL}}_{\mathrm{CNM}}\;\left(\text{days}\right)=\frac{1}{{\left(0.0088\;T+0.\;508\right)}^{2}}-\left\{\ln\left[-\ln\frac{\log N_{t}-\log N_{0}}{6.20-\log N_{0}}\right]-1\right\}\times \frac{6.20-\log N_{0}}{{\left(0.023\;T+0.15\right)}^{2}\times 2.7182}$$

(27) $${\mathrm{SL}}_{\text{Control}}\;\left(\text{days}\right)=\frac{1}{{\left(0.0677\;T+0.347\right)}^{2}}-\left\{\ln\left[-\ln\frac{\log N_{t}-\log N_{0}}{7.05-\log N_{0}}\right]-1\right\}\times \frac{7.05-\log N_{0}}{{\left(0.03\;T+0.20\right)}^{2}\times 2.7182}$$

Based on this SL expression, the estimated values of control, NM, and CNM Merguez stored at 7°C were 11.58, 20.69, and 30.32 days, respectively. The use of capsaicin nanoemulsion was found to be effective in extending the shelf life of Merguez 3 times compared to the control sample. This improvement is due to the antimicrobial activities of capsaicin, which inhibits bacterial spoilage, and retarding the oxidation and degradation of meat proteins and lipids.

## 4 Conclusion

In this study, capsaicin was efficiently encapsulated. It was shown that the type and proportion of almond gum, pea protein isolate, and citrus pectin had an impact on the mean droplet size, PDI, ξ‐potential, whiteness index, optical microscopy, plastic viscosity, capsaicin encapsulation yield, antioxidant activities (IC_50_), creaming index of the nanoemulsions. All tested responses were well described by different models as a function of proportions of almond gum, pea protein isolate, and citrus pectin. Besides, the rheological, antioxidant, and antibacterial properties were also examined. The obtained data revealed that all the nanoemulsions were non‐Newtonian fluids of pseudoplastic (shear thinning) behavior. The Casson model was the most suitable to describe this behavior. Furthermore, the nanoemulsions showed high antioxidant and antibacterial activities and were more sensitive against Gram‐positive bacteria. The conjugation process of almond gum, pea protein isolate, and pectin produced a more stable nanoemulsion. Based on optimal conditions, two nanoemulsions were prepared with and without capsaicin, used for Merguez preservation, and compared to the control sample. Therefore, the findings indicated the Merguez samples, with capsaicin nanoemulsion, enhanced their physicochemical characteristics, and textural properties. Interestingly, the shelf life of treated Merguez was increased by threefolds compared to the control sample. Furthermore, the aforementioned results proved that capsaicin encapsulation by the nanoemulsification had an important role in Merguez preservation which can be used in the enrichment of food products.

## Author Contributions

**Eya Soussi:** conceptualization (equal), data curation (equal), formal analysis (equal), methodology (equal), resources (equal), writing – original draft (equal). **Khouloud Rigane:** conceptualization (equal), data curation (equal), investigation (equal), methodology (equal), software (equal), writing – original draft (equal). **Anis Ben Hsouna:** data curation (equal), software (equal), validation (equal). **Miroslava Kačániová:** resources (equal), software (equal), validation (equal). **Wissem Mnif:** formal analysis (equal), funding acquisition (equal), supervision (equal), visualization (equal), writing – review and editing (equal). **Zaina Algarni:** formal analysis (equal), funding acquisition (equal), project administration (equal), software (equal). **Moncef Chouaibi:** conceptualization (equal), formal analysis (equal), methodology (equal), project administration (equal), resources (equal), supervision (equal), validation (equal), visualization (equal), writing – original draft (equal).

## Conflicts of Interest

The authors declare no conflicts of interest.

## Data Availability

The data that support the findings of this study are available on request from the corresponding author.
